# Wogonin increases gemcitabine sensitivity in pancreatic cancer by inhibiting Akt pathway

**DOI:** 10.3389/fphar.2022.1068855

**Published:** 2022-12-23

**Authors:** Tianli Zhang, Mengmeng Liu, Qing Liu, Gary Guishan Xiao

**Affiliations:** State Key Laboratory of Fine Chemicals, Department of Pharmaceutical Sciences, School of Chemical Engineering, Dalian University of Technology, Dalian, China

**Keywords:** wogonin, pancreatic cancer, gemcitabine, sensitization, drug resistance, mechanism

## Abstract

Pancreatic cancer has a high degree of malignancy and a low 5-year survival rate, and drug resistance is one of the main factors leading to poor prognosis of pancreatic cancer. Wogonin is a flavonoid drug isolated from Scutellaria baicalensis, which has certain antitumor activity. Hence the purpose of this study was to investigate whether wogonin can be used to enhance the sensitivity of pancreatic cancer to gemcitabine chemotherapy, and investigate its possible sensitization mechanism. *In vitro,* MTT assay showed that wogonin increased gemcitabine cytotoxicity in gemcitabine-resistant pancreatic cancer cells. *In vivo,* Wogonin combined with gemcitabine was found to inhibit tumor growth in orthotopic pancreatic cancer mouse model. In order to explore the sensitization mechanism, the differentially expressed genes (DEGs) of the gemcitabine-resistant cell line Panc-1 and the gemcitabine-sensitive cell line Bxpc-3 were screened through the GEO database, and 15 differentially expressed genes were obtained by intersecting with the potential targets of wogonin. Gene Ontology and KEGG enrichment analysis was performed. Bioinformatics results predicted that wogonin promoted pancreatic cancer cell apoptosis by inhibiting protein kinase B (Akt) signaling, thereby enhancing the sensitivity of gemcitabine to Pancreatic cancer. The above results were also verified by flow cytometry and Western blotting experiments. In conclusion, wogonin may enhance the sensitivity of gemcitabine by inhibiting Akt pathway.

## 1 Introduction

The rate of pancreatic cancer incidence in 2020 was 4.9 per 100000 for both sexes together, while mortality rate was 4.5 per 100000 from a global perspective (Ilic and Ilic, 2022). Pancreatic cancer is a malignant tumor with high degree of malignancy, difficult clinical detection and poor prognosis (Bray et al., 2018; Mcguigan et al., 2018; Park et al., 2021). The main risk factors associated with pancreatic cancer include factors such as alcoholism, smoking, obesity, diabetes, family history, and genetics (Ren and Wang, 2020; Hu et al., 2021). The early treatment of pancreatic cancer is mainly adjuvant gemcitabine chemotherapy after surgical resection, and the use of gemcitabine/nab-paclitaxel in the advanced treatment of pancreatic cancer can improve the poor prognosis to a certain extent (Neoptolemos et al., 2018). Although gemcitabine is used as a first-line drug for pancreatic cancer chemotherapy, it often leads to poor clinical outcomes due to the inherent drug resistance of pancreatic cancer cells (Yang et al., 2021). Therefore, for gemcitabine resistance, quercetin (Lee et al., 2015), Ginsenoside Rg3 (Zou et al., 2020) and Ursolic (Lin et al., 2020) has been reported to promote apoptosis of pancreatic cancer cells and enhance gemcitabine sensitivity. These findings prompted us to investigate whether other phytochemicals could sensitize pancreatic cancer cells to gemcitabine.

We selected one of the active ingredients wogonin according to the optimal toxicokinetic ADME rules (OB = 30.68% > 30% and DL = 0.23 > 0.18) (Liu et al., 2013) in the TCMSP database. Wogonin is a flavonoid compound extracted from the root of Scutellaria baicalensis (Banik et al., 2022). It has antioxidant activity, and anti-inflammatory, anti-tumor, immunomodulatory, neuroprotective effects (Huynh et al., 2020). Wogonin also acts as a chemosensitizer, reducing drug resistance in cancer therapy. When wogonin is used in combination with anticancer drugs such as etoposide, doxorubicin, 5-FU, and cisplatin (Huynh et al., 2017), it can induce tumor cell apoptosis (Wu et al., 2021) and protect normal cells from side effects.

Xing et al, (2019). Reported that wogonin enhanced the sensitivity of ovarian cancer cells to gemcitabine by inhibiting the PI3K/Akt signaling pathway. However, whether wogonin can enhance gemcitabine sensitivity and its sensitizing mechanism remain to be explored. Therefore, this study intends to preliminarily investigate whether wogonin has a sensitizing effect on the Pancreatic cancer by inhibiting the Akt signaling pathway.

## 2 Materials and methods

### 2.1 Materials and reagents

Wogonin (Cat. MB6663-20 mg) was purchased from Meilunbio (Dalian, China); Gemcitabine (Cat. PHR2582-50 mg) was purchased from Sigma Aldrich (St Louis, MO, United States); EDTA-free trypsin (C0207) was purchased from Beyotime Biotechnology (Shanghai, China); the anti-β-actin antibody (Cat. 66009-1-lg) was purchased from Proteintech (Rosemont, IL, United States) and antibodies against pAKT (Thr) antibody (Cat.ab131474), Akt (Cat.ab8805), BAD (Cat.ab32455) and Bcl-2 (Cat.ab32124) were purchased from Abcam (Massachusetts, United States). The anti-rabbit Ki67 (Cat. ab15580) was also purchased from Abcam.

### 2.2 Cell culture and MTT assays

Pancreatic cancer cell lines (PANC-1, BXPC-3, PANC-02) were purchased from the Peking Union Medical College Cell Bank (Beijing, China). All these cells were cultured in DMEM medium supplemented with 10% FBS. Panc-1 and Bxpc-3 cells were plated in 96-well plates, and treated with Gemcitabine or Wogonin for 72 h MTT assays. MTT was then performed as previously described (Zhang et al., 2022)

### 2.3 Annexin-V assay

The Annexin V-FITC Apoptosis Detection Kit (Cat. C1062S) was were purchased from the Beyotime Biotechnology (Shanghai, China). The Panc-1 cells were cultivated to the logarithmic growth phase, and treated with 10 μM, 20 μM, 40 μM, and 100 μM wogonin for 72 h, digested with EDTA-free trypsin, and then terminated with serum. Cells were washed with PBS twice and then esponded with 195 μl Annexin V-FITC binding solution, subsequently added 5 μl Annexin V-FITC and 10 μl PI for 10–20 min in the dark. Finally, flow cytometry was used to detect cell apoptosis. (PI and Annexin concentrations in the kit are inconvenient for the reagent supplier to provide)

### 2.4 Western blotting

Panc-1 cells were treated with 10, 20, 40, 100 μM Wogonin for 72 h. Total protein was extracted from the Panc-1 cells and from pancreatic tissue of Control group, Gem group and Gem + Wog group. Western blotting was then performed as previously described (Domenichini et al., 2019)

### 2.5 Animals

C57BL/6 mice were purchased from Liaoning Changsheng Biotechnology Co., Ltd. (Benxi, China). 2 × 10^6^ Mouse derived PANC-02 cells were injected into the pancreas tissue of male C57BL/6 mice to generate orthotopic pancreatic cancer mouse model. Eighteen mice were divided into three groups: Control group, Gemcitabine group (Gem group), and Gemcitabine Wogonin combined group (Gem + Wog group). Mice in the Gem group were intraperitoneally injected with gemcitabine (25 mgkg^−1^ i. g.) on the seventh and 14th days; Gem + Wog group were intraperitoneally injected with gemcitabine (25 mgkg^−1^ i. g.) on the seventh and 14th days, along with intragastric administration of Wogonin (50 mgkg^−1^ i. p.) in 7–21 days per day. Body weight were measured every 2 days. After 21 days, The mice were sacrificed and pancreas tumors were collected for mechanism research.

### 2.6 Histochemical staining

The collected pancreatic tumors were immersed in 4% paraformaldehyde, embedded in paraffin, and cut into small pieces. Pancreatic sections were then stained with Ki67 antibody according to the manufacturer’s instructions and simple images were obtained by using a light microscope at ×100 magnification (Zheng et al., 2020). IHC images were quantified by ImageJ.

### 2.7 Statistical analysis

The experimental data were analyzed using GraphPad Prism 8.0.1 statistical analysis software (GraphPad, San Diego, CA, United States). A two-tailed Student’s t-test or one-way analysis of variance (ANOVA) was used for statistical analyses. The following terminology was used to show statistical significance: *p < 0.05, **p < 0.01, ***p < 0.001.

### 2.8 Potential targets of wogonin

The active components of the drug can exert related biological functions through related targets. The active ingredients were screened in TCMSP (https://tcmsp-e.com/) according to the optimal toxicokinetic ADME rules. Wogonin (OB = 30.68, DL = 0.23) was obtained as possible core active components of pancreatic cancer. Genecards (https:// www.genecards.org/),SwissTargetPrediction (https://new.swisstargetprediction.ch/),BATMAN-TCM(http://bionet.ncpsb.org.cn/batman-tcm/),PharmMapper (Wang et al., 2016; Wang et al., 2017) (http://www.lilab-ecust.cn/pharmmapper/) were used to predict the potential targets of wogonin.

### 2.9 Identification of differentially expressed genes

GEO2R was used to identify DEGs between gemcitabine-resistant cell line Panc-1 and the gemcitabine-sensitive cell line Bxpc-3. A criteria of *p* < 0.05 and |logFC|≥2 were considered to be statistically significant. Then, the online software Venny 2.1.0 (http://bioinfogp.cnb.csic.es/tools/venny/index.html) was used to screen out overlapping DEGs. The Venn diagram was also drawn by Venny 2.1.0. (Song et al., 2020).

### 2.10 Construction of a “drug—targets—disease” network

The candidate targets of wogonin and protein markers of pancreatic cancer gemcitabine resistance-related genes were uploaded to IPA (Ingenuity Pathway Analysis, version 2019) for network and pathway analysis. IPA was used to construct pathways and networks based on interactions between genes and proteins. Through the “Compare” module in IPA, the pathways and networks involved in gemcitabine resistance-related genes and wogonin candidate targets in pancreatic cancer were identified.

### 2.11 Go and KEGG enrichment analysis

Metascape (https://metascape.org/gp/index.html#/main/step1) was used for GO and KEGG enrichment analysis. GO (Gene Ontology) terms are divided into CC (Cellular Component), biological process BP (Biological Process) and molecular function MF (Molecular Function). Set the filter condition MinOverlap = 3, *p* < 0.01.

## 3 Results

### 3.1 Wogonin increased gemcitabine cytotoxicity in gemcitabine-resistant pancreatic cancer cells

**FIGURE 1:**
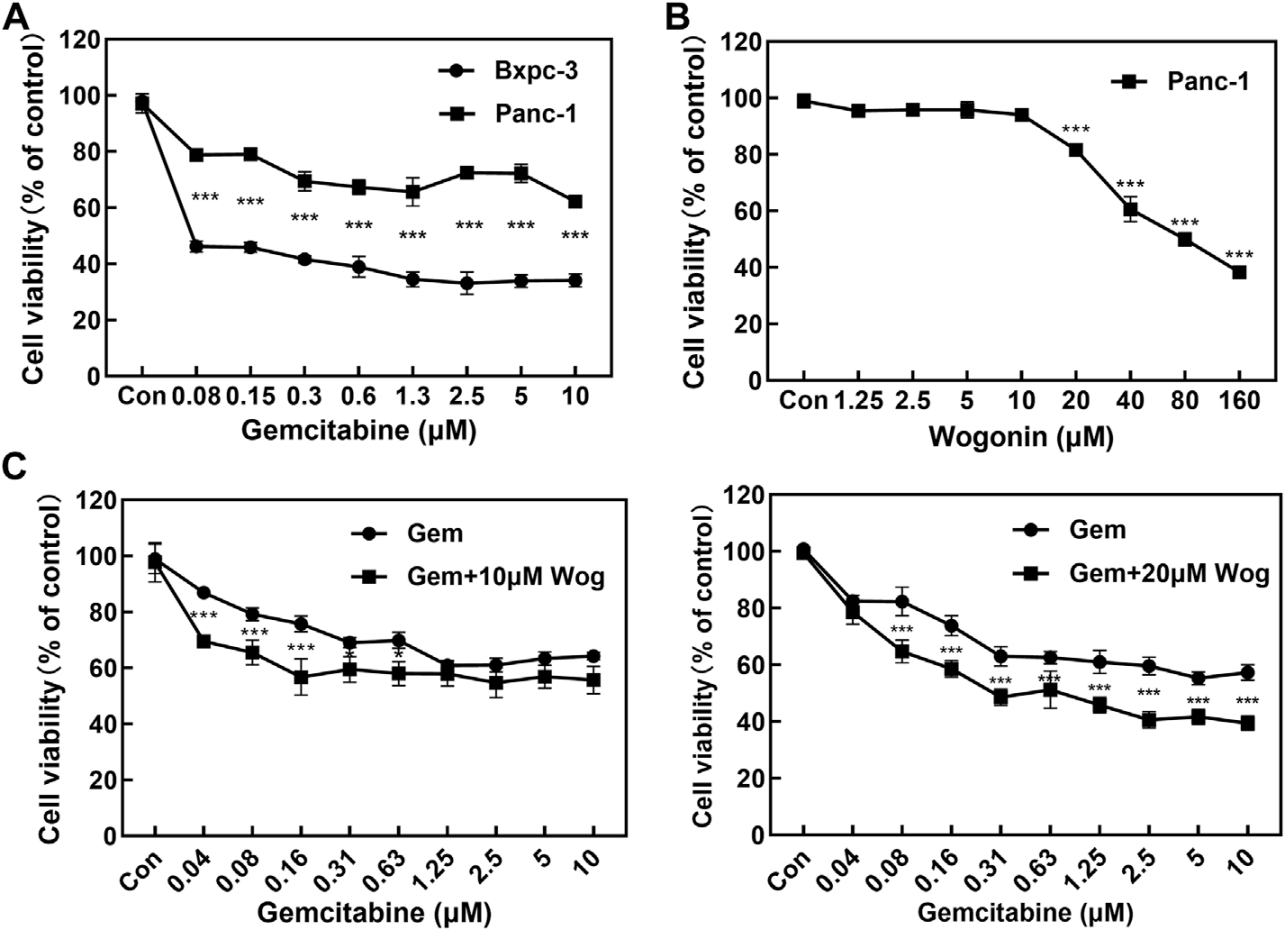
Wogonin combined with gemcitabine inhibits the proliferation of pancreatic cancer cells. **(A)** The cell viability of Panc-1 and Bxpc-3 cells treated with gemcitabine was assessed using MTT assay at 72 h. Compared with Bxpc-3, Gemcitabine displayed no significant antiproliferative effects to Panc-1 cell. **(B)** The cell viability of Panc-1 treated with wogonin was assessed using MTT assay at 72 h. The cell viability decreased with the elevated concentration of wogonin. **(C)** Sensitizing effect of wogonin in Panc-1 cells to gemcitabine. Cells were treated with gemcitabine (0, 0.04, 0.08, 0.15, 0.3, 0.6, 1.3, 2.5, 5 and 10 μM), Gem + Wog (10 μM) and Gem + Wog (20 μM) for 72 h and cell viability was determined by MTT assay. *p < 0.05, **p < 0.01, ***p < 0.001 vs. controls.

MTT assay was performed to assess the antiproliferative effects of pancreatic cancer cells by gemcitabine and wogonin treatment for 72 h. Panc-1 and Bxpc-3 pancreatic cancer cell lines were treated with gemcitabine at different concentrations (0, 0.08, 0.15, 0.3, 0.6, 1.3, 2.5, 5 and 10 μM) for 72 h. Panc-1 cell vitality decreased by 21.0%–37.7%, and Bxpc-3 cell viability decreased by 53.8%–66.9%, which displayed significant antiproliferative effects. (Figure 1A). As described in the literature (Cao et al., 2015), Panc-1 pancreatic cancer cell line is naturally resistant to gemcitabine, while Bxpc-3 pancreatic cancer cell line is sensitive to gemcitabine.

Gemcitabine-resistant pancreatic cancer cells Panc-1 was incubated with wogonin at different concentrations (0, 1.3, 2.5, 5, 10, 20, 40, 80, and 160 μM). Wogonin inhibited the growth of Panc-1 cells in dose-and time-dependent manners. MTT test showed that IC50 value of wogonin was 73.3 μM and the dose-effect curve exhibited that 10 μM wogonin displayed no significant antiproliferative effects on Panc-1 cells (survival rate was 94.1%); 20 μM wogonin displayed weak antiproliferative effects (survival rate was 78.7%) (Figure 1B)

10 and 20 μM wogonin were combined with different concentrations (0, 0.04, 0.08, 0.15, 0.3, 0.6, 1.3, 2.5, 5 and 10 μM) of gemcitabine for 72 h. As shown in Figure 1C, at gemcitabine concentrations of 0.04, 0.08 and 0.16 µM, 10 μM wogonin significantly enhanced sensitive of Panc-1 cells to gemcitabine (*p* < 0.001). At gemcitabine concentrations of 0.31 and 0.63 µM, Wogonin could slightly enhance the sensitivity of Panc-1 cells to gemcitabine (*p* < 0.05). When the concentration of wogonin was increased to 20 μM, combined with 0.08, 0.16, 0.31, 0.63, 1.25, 2.5, 5, 10 μM of gemcitabine could further significantly inhibit the proliferation of Panc-1 cells. These results showed that the wogonin could significantly increase gemcitabine cytotoxicity in gemcitabine-resistant pancreatic cancer cells.

### 3.2 Prediction of targets for wogonin sensitization

**FIGURE 2:**
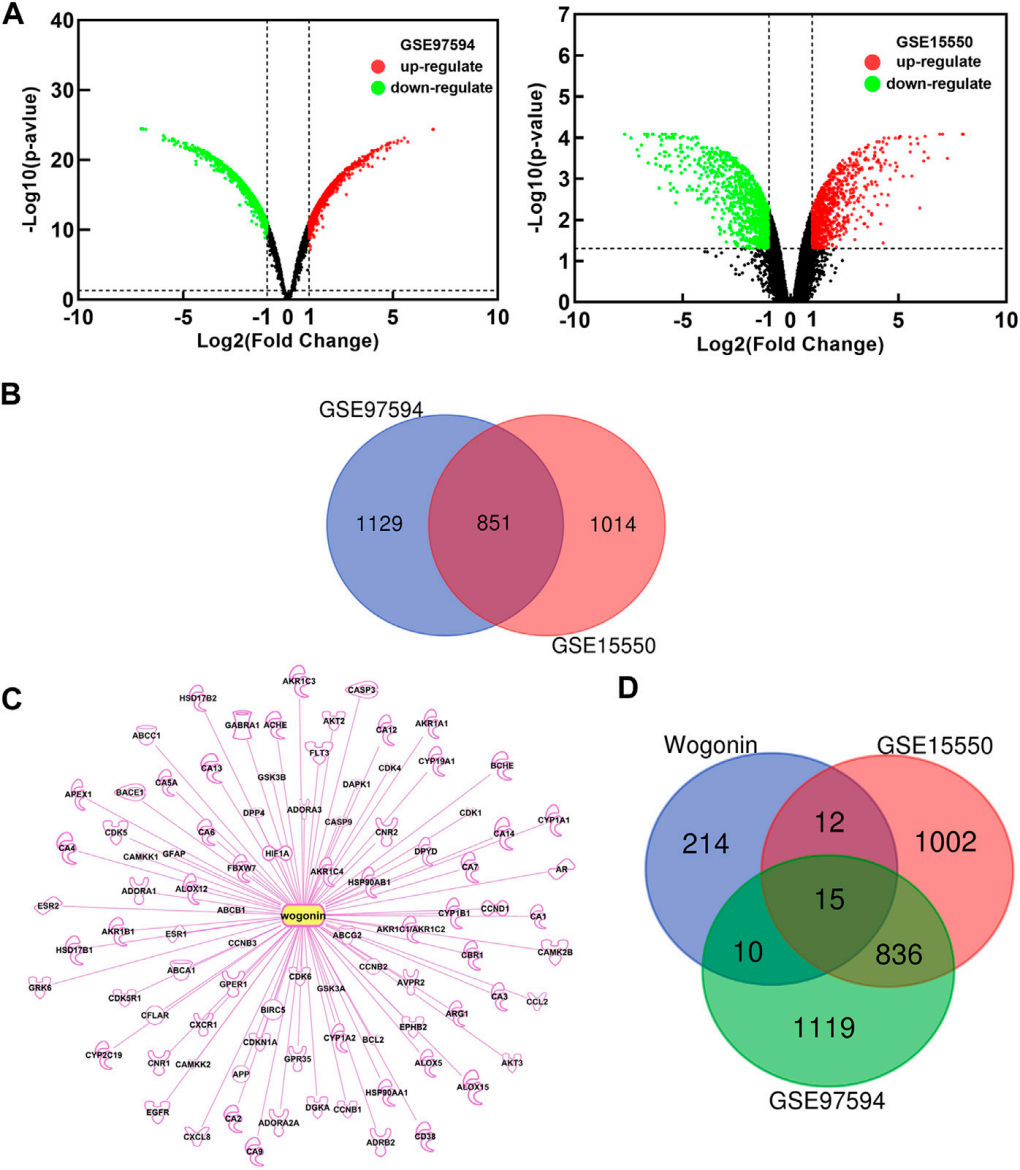
Potential target of wogonin to enhance gemcitabine sensitivity. **(A)**Diferential genes volcano map analyzed by GEO chips. Global gene expression of gemcitabine-resistant cell line Panc-1 vs gemcitabine-sensitive cell line Bxpc-3 **(B)** Venn diagram of 851 DEGs related to gemcitabine resistance **(C)** Potential targets of wogonin **(D)** Venn diagram of 15 DEGs related to wogonin sensitization in gemcitabine-resistant pancreatic cancer.

In order to explore the possible mechanism of wogonin sensitization, two gene chips (GSE15550, GSE97594) were found in the GEO database. By comparing the gemcitabine-resistant cell line Panc-1 and the gemcitabine-sensitive cell line Bxpc-3, differentially expressed genes (DEGs) related to gemcitabine resistance in pancreatic cancer were obtained. Based on the criteria of *p* < 0.05 and |logFC|≥2, a total of 1865 DEGs were obtained in GSE15550, including 917 up-regulated genes and 948 down-regulated genes. In gene chip GSE97594, 1980 DEGs were identified, including 1064 up-regulated genes and 916 down-regulated genes. The DEGs were used to build a volcano map (Figure 2A) Venn 2.1.0 was used to screen for common DEGs between GSE15550 and GSE97594. The obtained 851 genes were differentially expressed genes (DEGs) related to gemcitabine resistance (Figure 2B). Through GeneCards, TCMSP, SwissTargetPrediction, BATMAN-TCM, PharmMapper and IPA databases, 251 potential targets related to wogonin were obtained (Figure 2C). Potential targets of wogonin were intersected with 851 DEGs, and 15 DEGs were obtained (Figure 2D). These fifteen DEGs, including AKT2, CCL2, HSP90AA1, PDE5A, PTGS1, BCHE, SERPINB5, CA2, SRC, DGKA, HIF1A, PTGS2, ABCA1, DPYD, and AKR1C3 were potential target of wogonin to enhance gemcitabine sensitivity in pancreatic cancer.

### 3.3 Prediction of the sensitization mechanism of wogonin

**FIGURE 3:**
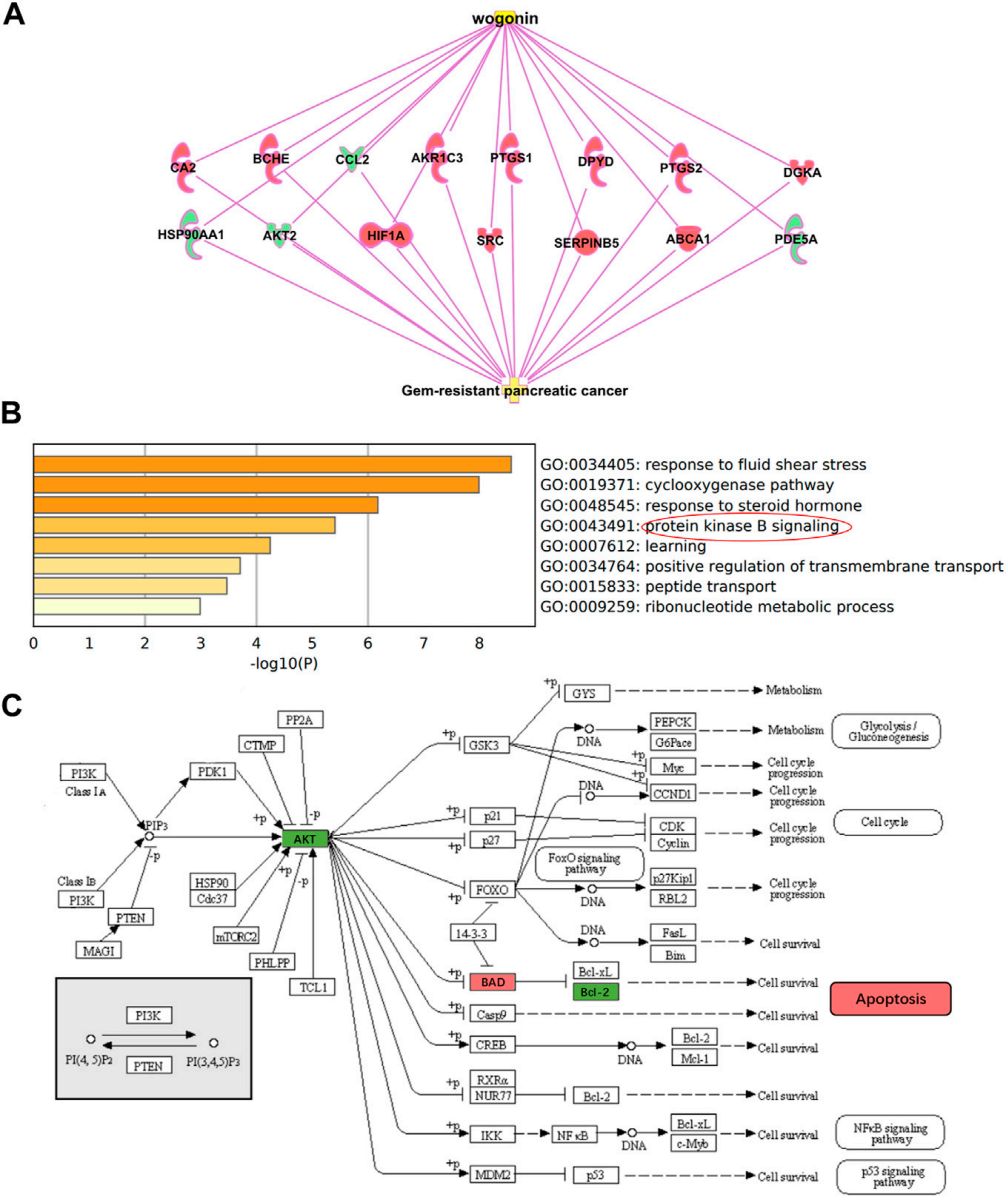
Potential mechanism of wogonin sensitization **(A)** “wogonin-target-gemcitabine resistance” network map. Wogonin can enhance gemcitabine sensitivity by downregulating AKT2. **(B)** Go enrichment analysis of 15 DEGs, which were enriched in protein kinase B (Akt) signaling. **(C)** Hypothesis on the sensitization mechanism of wogonin to gemcitabine in pancreatic cancer.

Through IPA (Ingenuity Pathway Analysis) software, a “wogonin-target-gemcitabine resistance” network map was constructed. Wogonin may enhance the sensitivity of gemcitabine in pancreatic cancer by inhibiting AKT2, CCL2, HSP90AA1, PDE5A, or activating PTGS1, BCHE, SERPINB5, CA2, SRC, DGKA, HIF1A, PTGS2, ABCA1, DPYD and AKR1C3 (Figure 3A).

GO and KEGG enrichment analysis for the 15 DEGs were performed using the Metascape. The enriched GO terms were divided into CC, BP, and MF ontologies. The results of GO analysis indicated that DEGs were mainly enriched in BPs, including response to fluid shear stress, cyclooxygenase pathway, response to steroid hormone, protein kinase B signaling, learning, positive regulation of transmembrane transport, peptide transport, and ribonucleotide metabolic process (Figure 3B). MF analysis showed that the DEGs were significantly enriched in heme binding, phosphotransferase activity, ubiquitin protein ligase binding, protein homodimerization activity, and hydrolase activity. For the cell component, the DEGs were enriched in membrane raft and axon. In addition, the results of KEGG pathway analysis showed that DEGs were mainly enriched in pathways in Fluid shear stress and atherosclerosis, Regulation of lipolysis in adipocytes, IL-17 signaling pathway, and Choline metabolism in cancer.

As shown in Figure 2A, wogonin can down-regulate the expression of AKT2 and enhance the sensitivity of gemcitabine to pancreatic cancer cell; As shown in Figure 3B, the differentially expressed genes for wogonin sensitization were enriched in protein kinase B (Akt) signaling. In summary, we selected protein kinase B (Akt) signaling pathway (GO:0043491). Through the KEGG website (https://www.kegg.jp/), we can hypothesize that wogonin can promote the apoptosis of gemcitabine-resistant cell lines by inhibiting Akt and its downstream apoptosis-related proteins BAD and Bcl-2 (Figure 3C), and was verified by follow-up experiments.

### 3.4 Wogonin promoted apoptosis of Panc-1 cells by inhibiting Akt pathway

**FIGURE 4:**
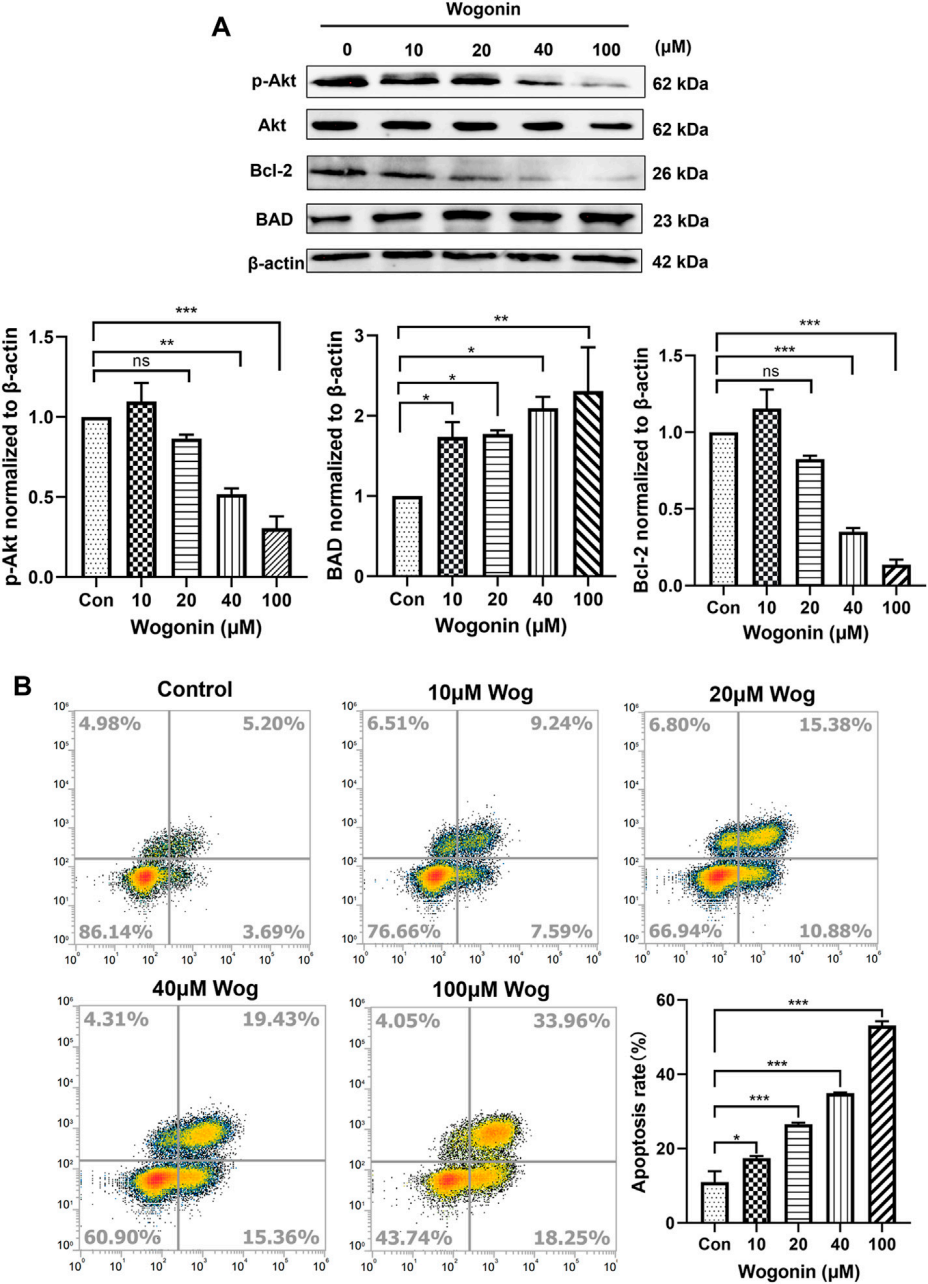
Wogonin promoted apoptosis of Panc-1 cells by inhibiting Akt pathway **(A)**gemcitabine-resistant cell line Panc-1 was treated with 10, 20, 40 and 100 μM wogonin for 72 h, then the protein levels of Akt, p-Akt, BAD and Bcl-2 were detected by western blot. **(B)**Flow cytometry was used to detect the apoptosis of Panc-1 cells treated with different concentrations of wogonin for 72 h *p < 0.05, **p < 0.01, ***p < 0.001 vs. controls.

The expression of Akt pathway was verified by WB experiment. The gemcitabine-resistant pancreatic cell line Panc-1 was selected, and wogonin at final concentrations of 10, 20, 40, and 100 μM was added. After 72 h of incubation, the cell protein was extracted and the expression of Akt, p-Akt, BAD, Bcl-2 was detected. As shown in Figure 4A, with the increase of wogonin concentration, the protein expression of Akt remained unchanged, while the protein expression of p-Akt decreased significantly, and the protein expression of the pro-apoptotic gene Bad increased. The protein expression of the anti-apoptotic gene Bcl-2 decreased, which was consistent with the bioinformatics conclusions.

Cell apoptosis was detected by flow cytometry. In Panc-1 cells, 10, 20, 40, 100 μM wogonin was added, and the apoptosis of Panc-1 was detected after 72 h of incubation. Annexin V-FITC and PI double staining positive means late apoptotic cells. Under the action of 10 μM wogonin, it had a certain promotion effect on the apoptosis of Panc-1 (*p* < 0.05), and under the action of 20, 40 and 100 μM wogonin, it significantly promoted the late apoptosis of cells (*p* < 0.001), which was consistent with Western blot results (Figure 4B). We can conclude that wogonin can promote the apoptosis of Gemcitabine-resistant pancreatic cell line Panc-1 by inhibiting Akt signaling.

### 3.5 Wogonin inhibitd pancreatic cancer *in vivo* by inhibiting Akt pathway

**FIGURE 5:**
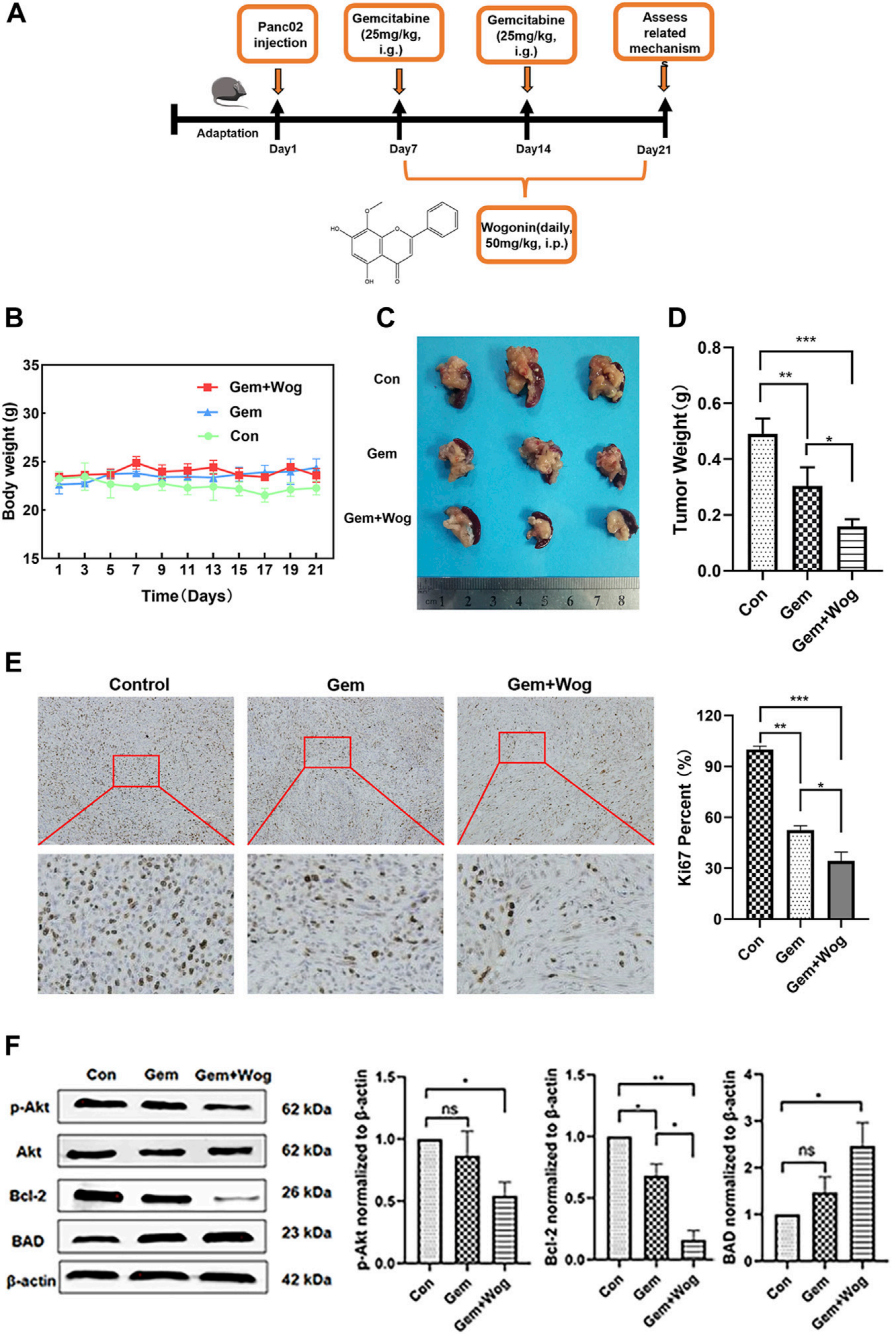
Wogonin inhibitd pancreatic cancer *in vivo* by inhibiting Akt pathway **(A)**Orthotopic pancreatic cancer mouse model experimental design **(B)** Body weight changes of mice in Con group, Gem group and Gem + Wog group within 21 days **(C)** Tumor size **(D)** Tumor weight **(E)** The fixed sections of mouse pancreas and pancreatic cancer were stained with Ki67 to detect tumor proliferation **(F)** After cryopreservation of mouse pancreatic tumors, Western Blot was detected to compare the expression of p-AKT, AKT, BAD and bcl-2 proteins in Con group, Gem group and Gem + Wog group. *p < 0.05, **p < 0.01, * **p < 0.001 vs. controls.

In order to explore the sensitization effect of wogonin on gemcitabine in pancreatic cancer, we generated orthotopic pancreatic cancer mouse model in C57BL/6 mice and administered the drugs on time. The experimental design is shown in the timeline (Figure 5A) and in the method of animals. As shown in Figure 5B, compared with the Control group, the body weight of the mice in the Gem group (25 mg/kg) and the Gem + Wog group (25 mg/kg Gem +50 mg/kg Wog) had no downward trend. Compared with the Control group, the tumor size of Gem + Wog group was significantly reduced (*p* < 0.05) (Figure 5D). The tumor inhibition rates in Gem group and Gem + Wog group were 26.2% and 59.6%, respectively, and the tumor inhibition rate in the Gem + Wog group was significantly higher than that in the Gem group. The combination of wogonin and gemcitabine can inhibit the growth of pancreatic in tumor, and the effect is better than that of gemcitabine alone. Ki67 staining indicated tissue proliferation (Kreipe, 2018). Gem + Wog group significantly reduced the degree of tissue malignancy (Figure 5E). As shown in Figure 5F, the expression of p-Akt protein decreased, the expression of its downstream pro-apoptotic gene BAD increased, and the expression of anti-apoptotic gene Bcl-2 decreased, which was consistent with the *in vitro* experiments. The results showed that wogonin combined with gemcitabine inhibited the Akt signaling pathway *in vivo*, and inhibited the growth of orthotopic pancreatic cancer.

## 4 Discussion

Wogonin can induce senescence of breast cancer cells (Yang et al., 2020) and promote apoptosis of cervical cancer (Kim et al., 2013) and gastric cancer cells (Hong et al., 2018). Research shows that wogonin can cooperate with chemotherapy drugs such as docetaxel (Wang et al., 2018) and cisplatin (Xu et al., 2021) to achieve better anti-tumor effect. In this study, through bioinformatics analysis and experimental verification, it is showed that wogonin may enhance gemcitabine sensitivity in pancreatic cancer.

Wogonin can inhibit tumor proliferation and invasion (Liu et al., 2016). Through the MTT assay, it was demonstrated that wogonin inhibited the proliferation of pancreatic cancer cells. At the concentration of 10 μM, wogonin could also sensitize Panc-1 cell to gemcitabine (Figure 1).

In order to explore the sensitization mechanism of wogonin to gemcitabine, the bioinformatics method was used to predict potential target. Bioinformatics prediction showed that the sensitizing target of wogonin is associated with AKT2, and GO analysis indicated that DEGs are inriched in protein kinase B (Akt) signaling (Figure 2, Figure 3). According to Yu et al, (2010)expression of the apoptosis-related genes Bcl-2 and BAD are associated with chemosensitivity. BAD and Bcl-2 are also potential target of wogonin in glioma (Wang et al., 2021). The results of flow cytometry and Western blot showed that wogonin may enhance the sensitivity of pancreatic cancer cells to gemcitabine by inhibiting p-Akt, anti-apoptotic gene Bcl-2, activating pro-apoptotic gene BAD, and promoting apoptosis of Panc-1 (Figure 4, Figure 5).

Meanwhile, bioinformatics predicted that wogonin could enhance gemcitabine sensitivity of pancreatic cancer by inhibiting AKT2, CCL2, HSP90AA1, PDE5A, and activating PTGS1, BCHE, SERPINB5, CA2, SRC, DGKA, HIF1A, PTGS2, ABCA1, DPYD, AKR1C3. According to the literature, AKT2 (Chen et al., 2012), CCL2 (Monti et al., 2004), HSP90 (Ghadban et al., 2017), CA2 (Zhao et al., 2021), SRC (Lin et al., 2019), HIF1A (Xu et al., 2021), ABCA1 (Shen and Yan, 2021), DPYD (Delhorme et al., 2022) and AKR1C3 (Phoo et al., 2021) are associated with drug resistance. The biological process of wogonin sensitization to gemcitabine includes: response to fluid shear stress, cyclooxygenase pathway, response to steroid hormones, positive regulation of transmembrane transport, peptide transport, and ribonucleoside acid metabolism process (Figure 3). And the results would be more reliable if gemcitabine resistant cells of the Pancreatic cancer were constructed and the Orthotopic pancreatic cancer mouse model was constructed. This study has not been experimentally verified, and further research is needed.

## 5 Conclusion

Our study demonstrates that wogonin inhibits the proliferation of Pancreatic cancer cells both *in vivo* and *invitro*. Through bioinformatics analysis and experimental verification, the mechanism of wogonin sensitizing gemcitabine in pancreatic cancer may be through inhibition of Akt pathway. Therefore, wogonin may be a novel drug with sensitizing effect in pancreatic cancer.

## Data Availability

The raw data supporting the conclusion of this article will be made available by the authors, without undue reservation.
