# Supplementary material for: Wogonin increases gemcitabine sensitivity in pancreatic cancer by inhibiting Akt pathway
Source: Front Pharmacol. 2022 Dec 23;13:1068855. doi: 10.3389/fphar.2022.1068855 (PMC9816391; doi:10.3389/fphar.2022.1068855)
Supplement: Supplementary file 1 [file DataSheet1.ZIP › flow cytometry.pdf]

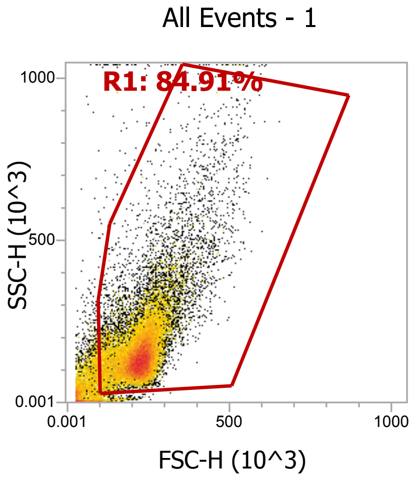

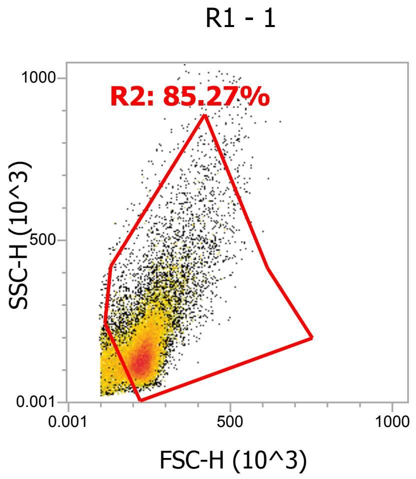

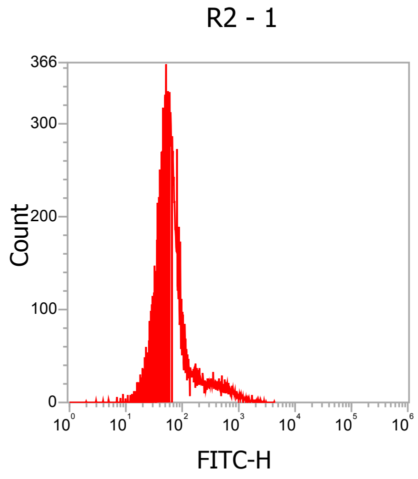

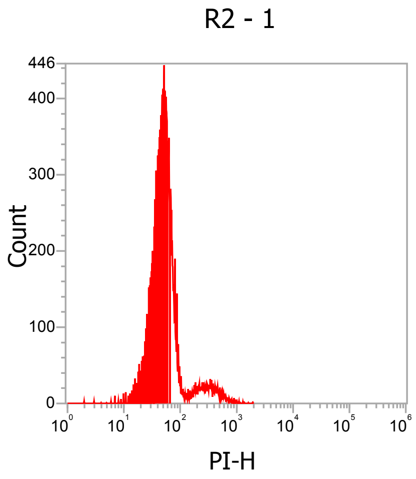

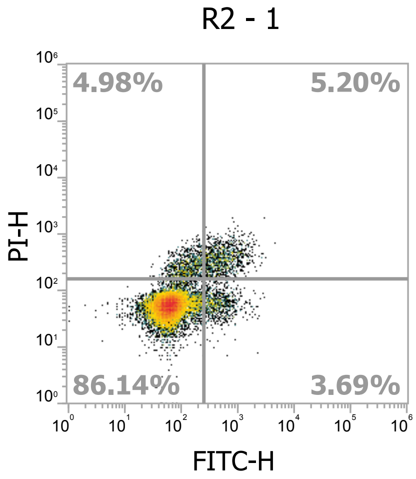

Parameters: **FITC - BL1-H vs PI - YL1-H**
Gate: **R2**
Experiment: **20211103-xgs**
Group: **Group**
Sample: **1**
Time Recorded: **15:16:35**

Name	Gate	X Parameter	Y Parameter	Count	%Total	%Gated	X Mean
All Events	All Events	N/A	N/A	27,205	100.00	100.00	N/A
R1	R1	FSC - FSC-H	SSC - SSC-H	23,101	84.91	84.91	229,783
R2	R2	FSC - FSC-H	SSC - SSC-H	19,698	72.41	85.27	242,733
FITC-/PI+	FITC-/PI+	FITC - BL1-H	PI - YL1-H	980	3.60	4.98	139
FITC+/PI+	FITC+/PI+	FITC - BL1-H	PI - YL1-H	1,024	3.76	5.20	660
FITC-/PI-	FITC-/PI-	FITC - BL1-H	PI - YL1-H	16,967	62.37	86.14	68
FITC+/PI-	FITC+/PI-	FITC - BL1-H	PI - YL1-H	727	2.67	3.69	549

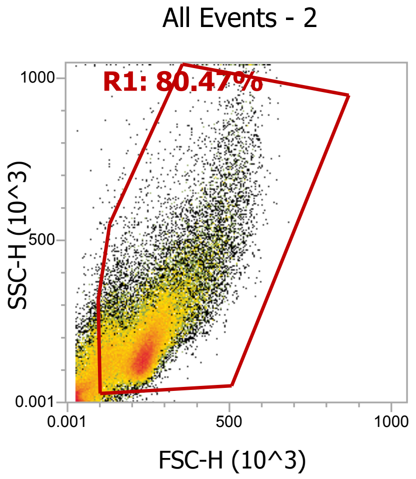

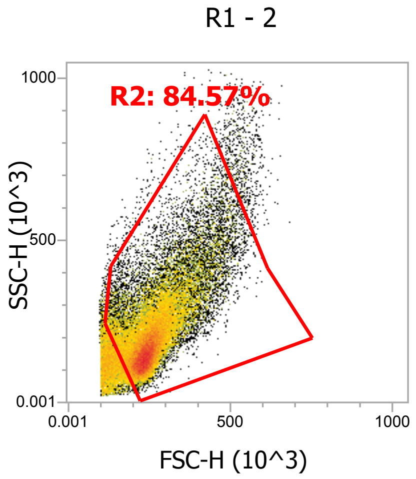

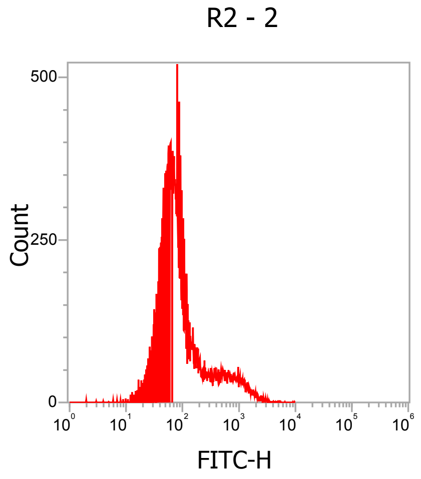

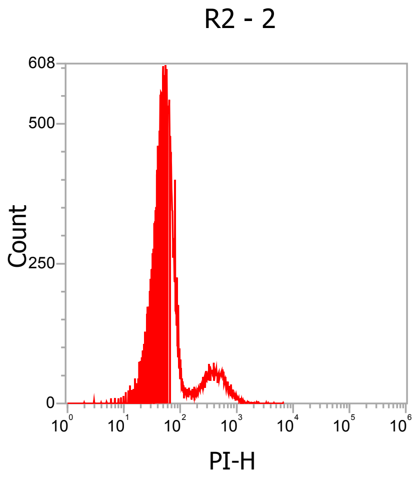

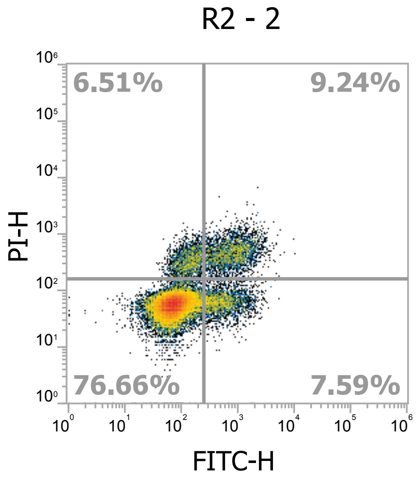

Parameters: **FITC - BL1-H vs PI - YL1-H**
Gate: **R2**
Experiment: **20211103-xgs**
Group: **Group**
Sample: **2**
Time Recorded: **15:22:57**

Name	Gate	X Parameter	Y Parameter	Count	%Total	%Gated	X Mean
All Events	All Events	N/A	N/A	47,900	100.00	100.00	N/A
R1	R1	FSC - FSC-H	SSC - SSC-H	38,546	80.47	80.47	253,554
R2	R2	FSC - FSC-H	SSC - SSC-H	32,600	68.06	84.57	267,247
FITC-/PI+	FITC-/PI+	FITC - BL1-H	PI - YL1-H	2,123	4.43	6.51	144
FITC+/PI+	FITC+/PI+	FITC - BL1-H	PI - YL1-H	3,013	6.29	9.24	969
FITC-/PI-	FITC-/PI-	FITC - BL1-H	PI - YL1-H	24,991	52.17	76.66	79
FITC+/PI-	FITC+/PI-	FITC - BL1-H	PI - YL1-H	2,473	5.16	7.59	690

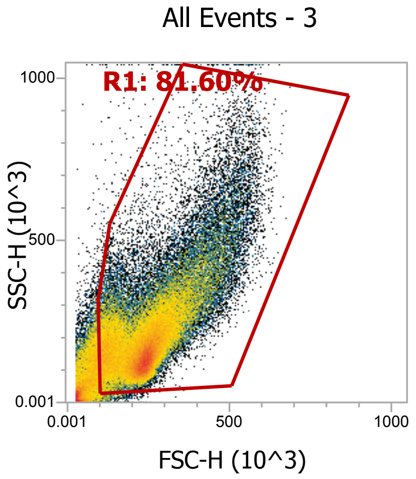

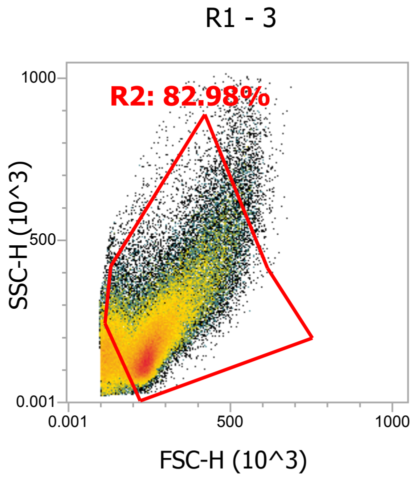

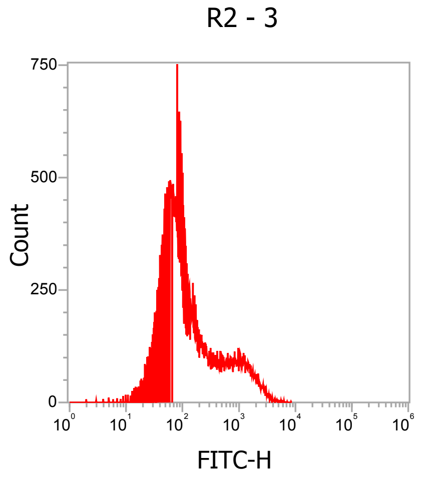

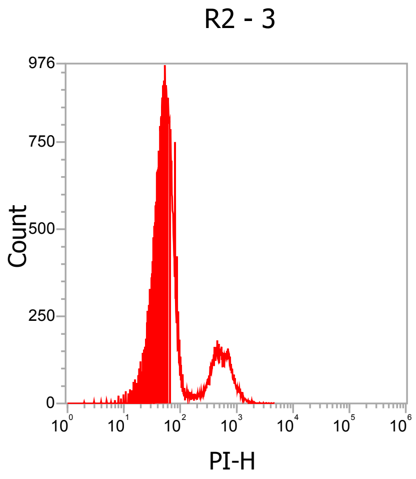

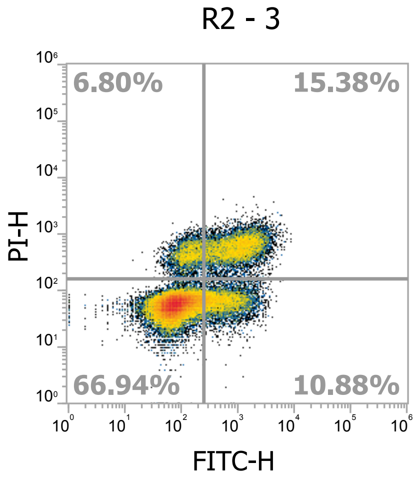

Parameters: **FITC - BL1-H vs PI - YL1-H**
Gate: **R2**
Experiment: **20211103-xgs**
Group: **Group**
Sample: **3**
Time Recorded: **15:24:35**

Name	Gate	X Parameter	Y Parameter	Count	%Total	%Gated	X Mean
All Events	All Events	N/A	N/A	83,290	100.00	100.00	N/A
R1	R1	FSC - FSC-H	SSC - SSC-H	67,961	81.60	81.60	260,999
R2	R2	FSC - FSC-H	SSC - SSC-H	56,393	67.71	82.98	277,460
FITC-/PI+	FITC-/PI+	FITC - BL1-H	PI - YL1-H	3,836	4.61	6.80	146
FITC+/PI+	FITC+/PI+	FITC - BL1-H	PI - YL1-H	8,672	10.41	15.38	1,246
FITC-/PI-	FITC-/PI-	FITC - BL1-H	PI - YL1-H	37,748	45.32	66.94	90
FITC+/PI-	FITC+/PI-	FITC - BL1-H	PI - YL1-H	6,137	7.37	10.88	749

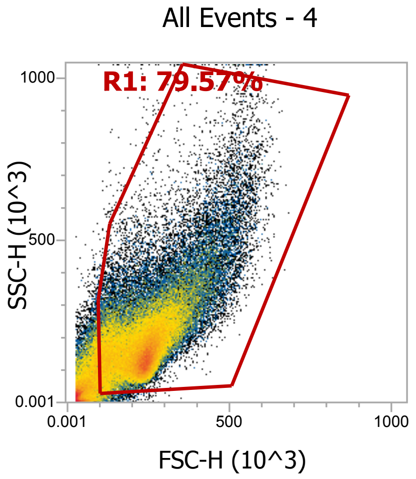

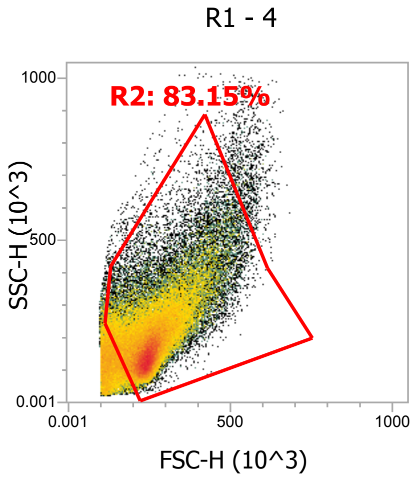

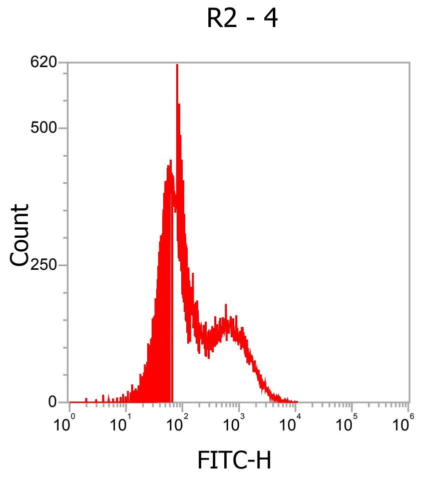

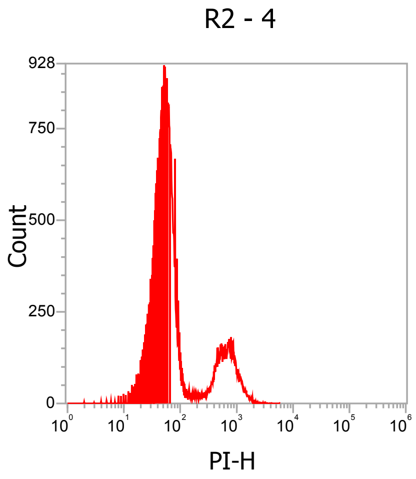

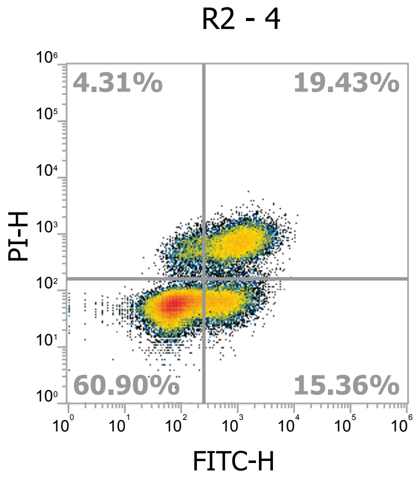

Parameters: **FITC - BL1-H vs PI - YL1-H**
Gate: **R2**
Experiment: **20211103-xgs**
Group: **Group**
Sample: **4**
Time Recorded: **15:26:58**

Name	Gate	X Parameter	Y Parameter	Count	%Total	%Gated	X Mean
All Events	All Events	N/A	N/A	84,421	100.00	100.00	N/A
R1	R1	FSC - FSC-H	SSC - SSC-H	67,178	79.57	79.57	255,487
R2	R2	FSC - FSC-H	SSC - SSC-H	55,859	66.17	83.15	272,466
FITC-/PI+	FITC-/PI+	FITC - BL1-H	PI - YL1-H	2,406	2.85	4.31	152
FITC+/PI+	FITC+/PI+	FITC - BL1-H	PI - YL1-H	10,855	12.86	19.43	1,280
FITC-/PI-	FITC-/PI-	FITC - BL1-H	PI - YL1-H	34,020	40.30	60.90	91
FITC+/PI-	FITC+/PI-	FITC - BL1-H	PI - YL1-H	8,578	10.16	15.36	688

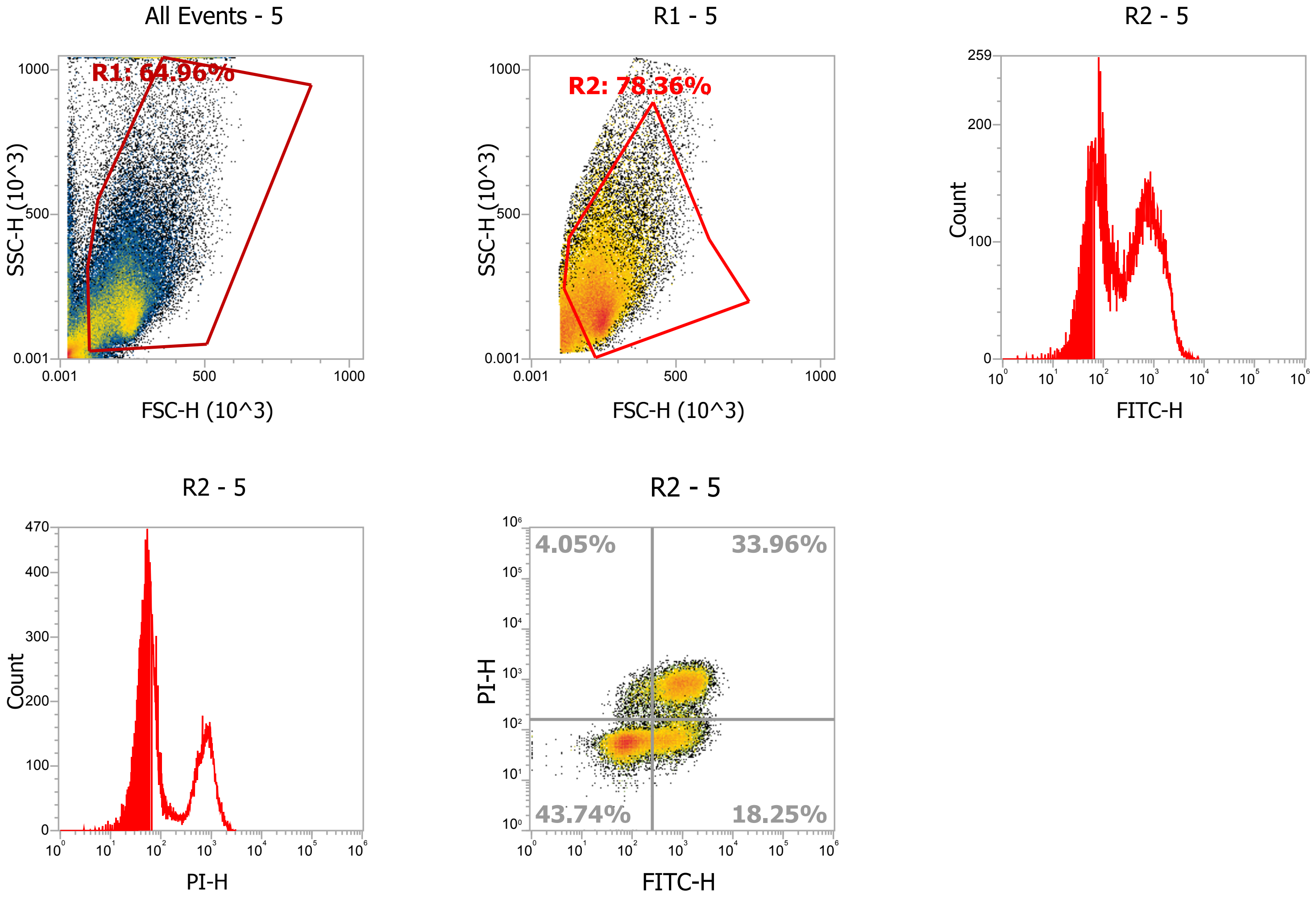

Parameters: **FITC - BL1-H vs PI - YL1-H**
Gate: **R2**
Experiment: **20211103-xgs**
Group: **Group**
Sample: **5**
Time Recorded: **15:28:31**

Name	Gate	X Parameter	Y Parameter	Count	%Total	%Gated	X Mean
All Events	All Events	N/A	N/A	65,728	100.00	100.00	N/A
R1	R1	FSC - FSC-H	SSC - SSC-H	42,694	64.96	64.96	232,522
R2	R2	FSC - FSC-H	SSC - SSC-H	33,454	50.90	78.36	251,717
FITC-/PI+	FITC-/PI+	FITC - BL1-H	PI - YL1-H	1,354	2.06	4.05	155
FITC+/PI+	FITC+/PI+	FITC - BL1-H	PI - YL1-H	11,360	17.28	33.96	1,172
FITC-/PI-	FITC-/PI-	FITC - BL1-H	PI - YL1-H	14,633	22.26	43.74	96
FITC+/PI-	FITC+/PI-	FITC - BL1-H	PI - YL1-H	6,107	9.29	18.25	791
