# Supplementary figures and images for: Wogonin increases gemcitabine sensitivity in pancreatic cancer by inhibiting Akt pathway
Source: Front Pharmacol. 2022 Dec 23;13:1068855. doi: 10.3389/fphar.2022.1068855 (PMC9816391; doi:10.3389/fphar.2022.1068855)

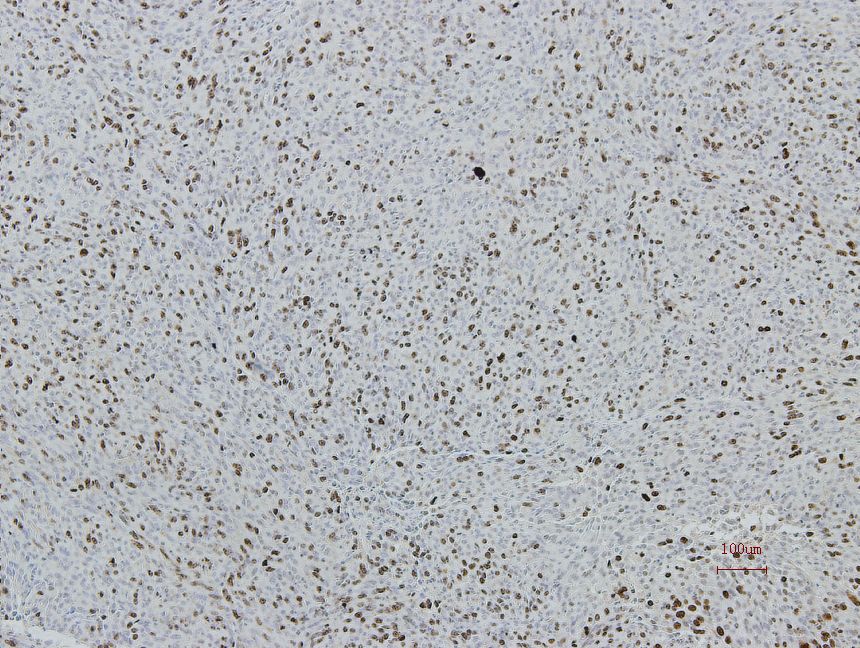

Supplement: Supplementary file 1 [file DataSheet1.ZIP › Ki67-Control.JPG]

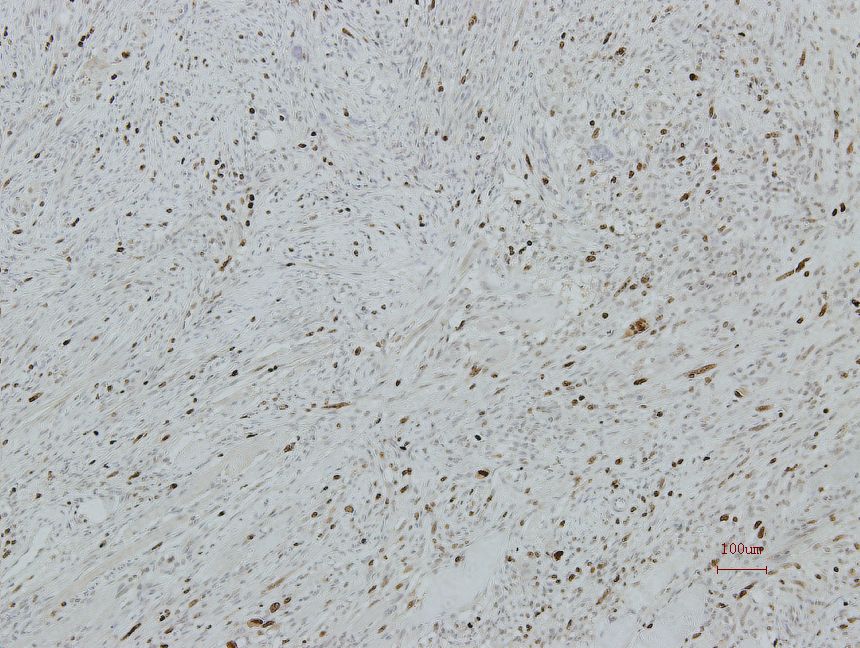

Supplement: Supplementary file 1 [file DataSheet1.ZIP › Ki67-Gem+Wog.JPG]

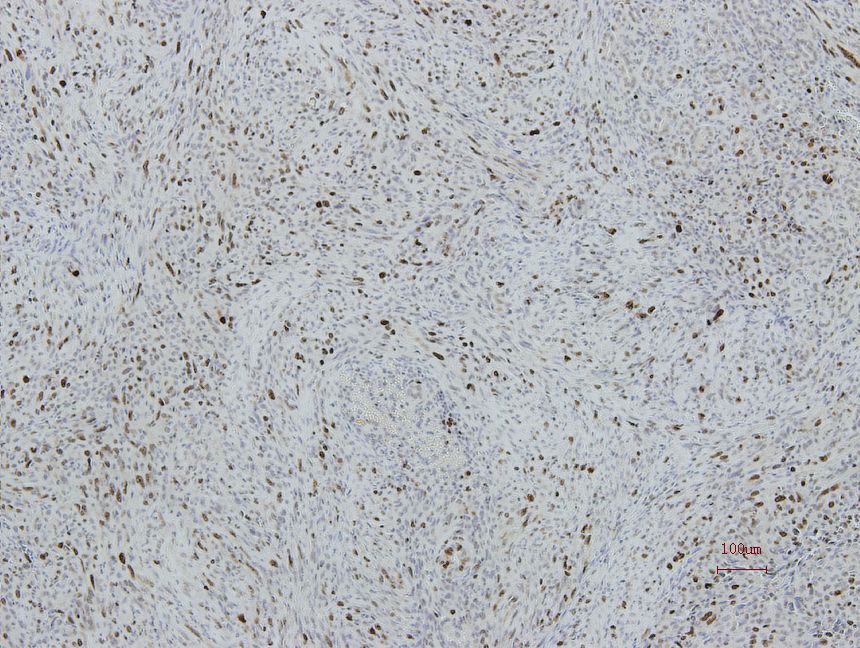

Supplement: Supplementary file 1 [file DataSheet1.ZIP › Ki67-Gem.JPG]

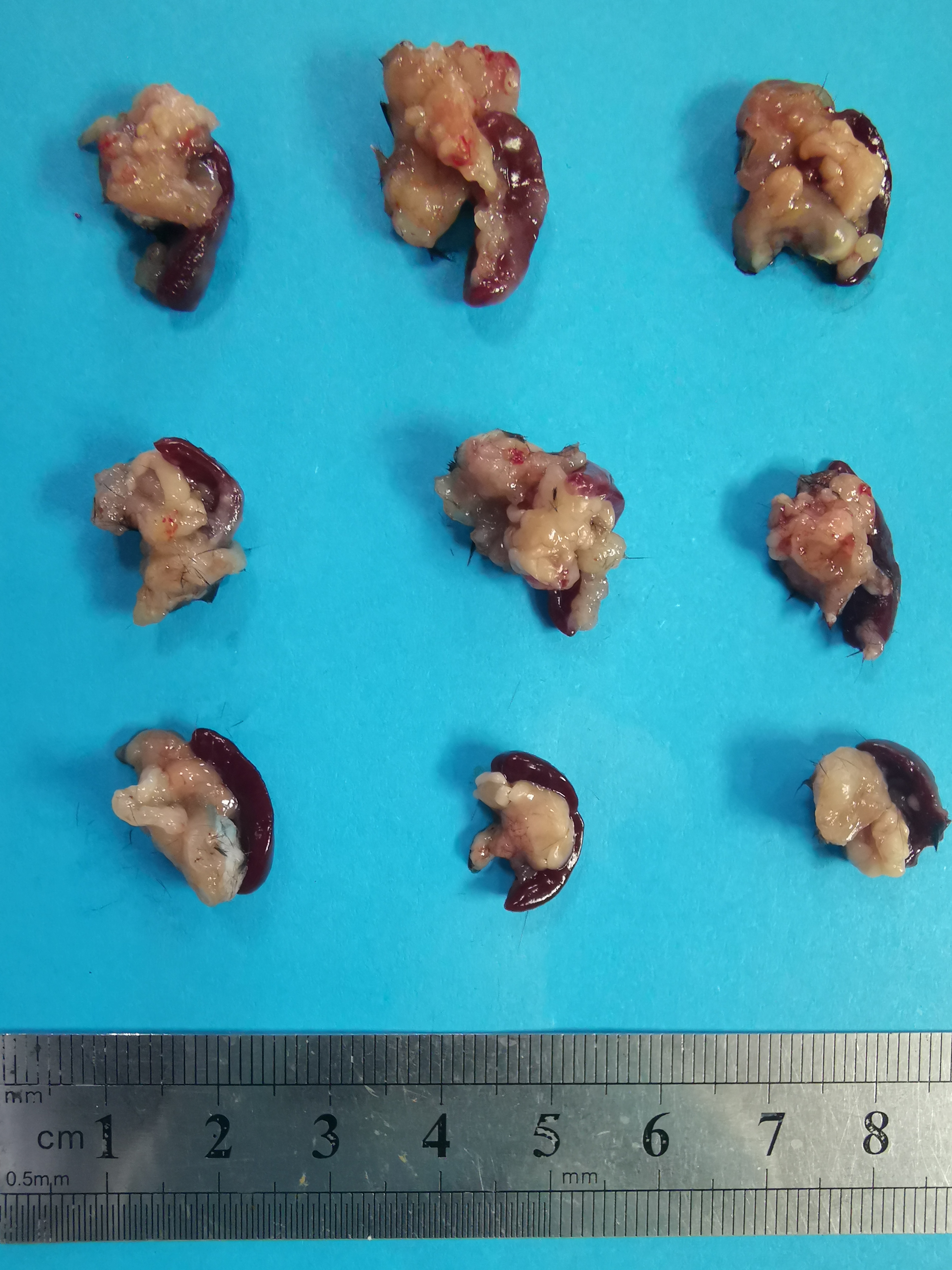

Supplement: Supplementary file 1 [file DataSheet1.ZIP › tumor.jpg]

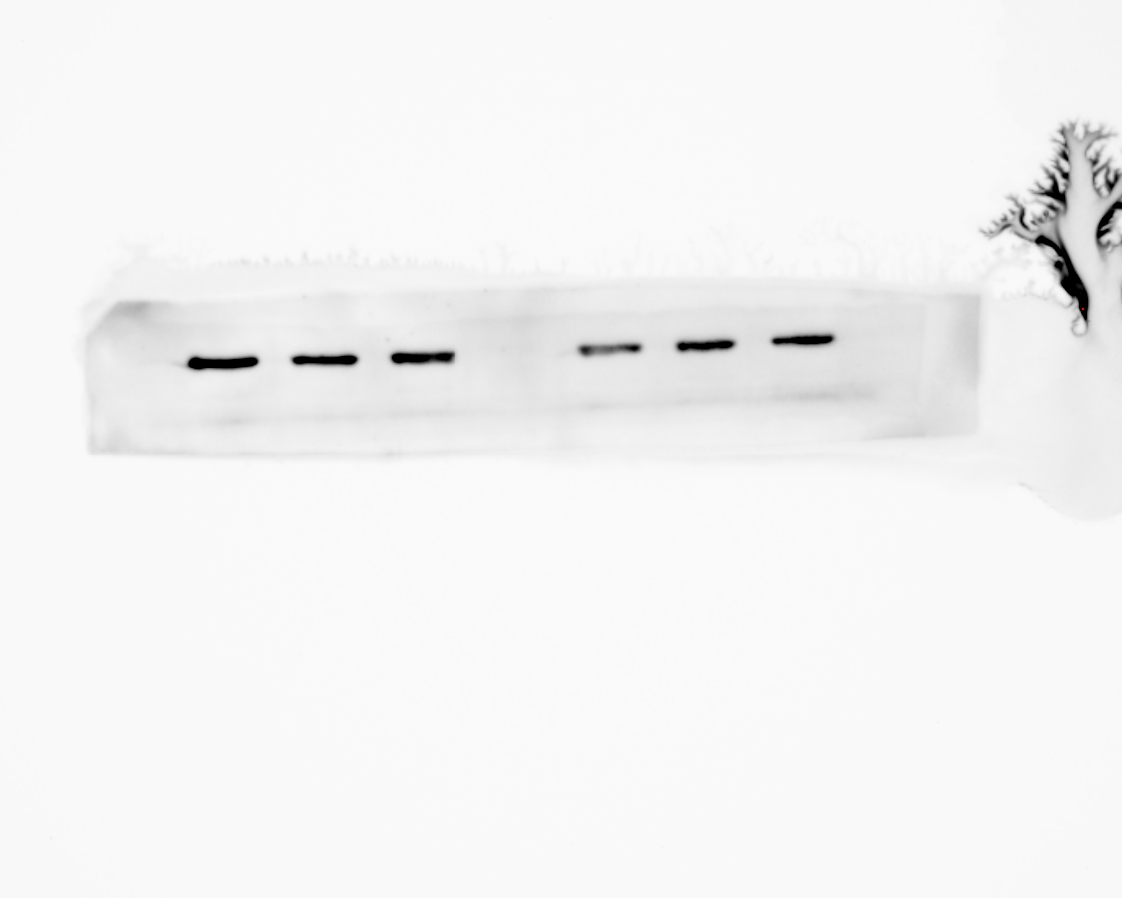

Supplement: Supplementary file 1 [file DataSheet1.ZIP › WB-mouse Akt.jpg]

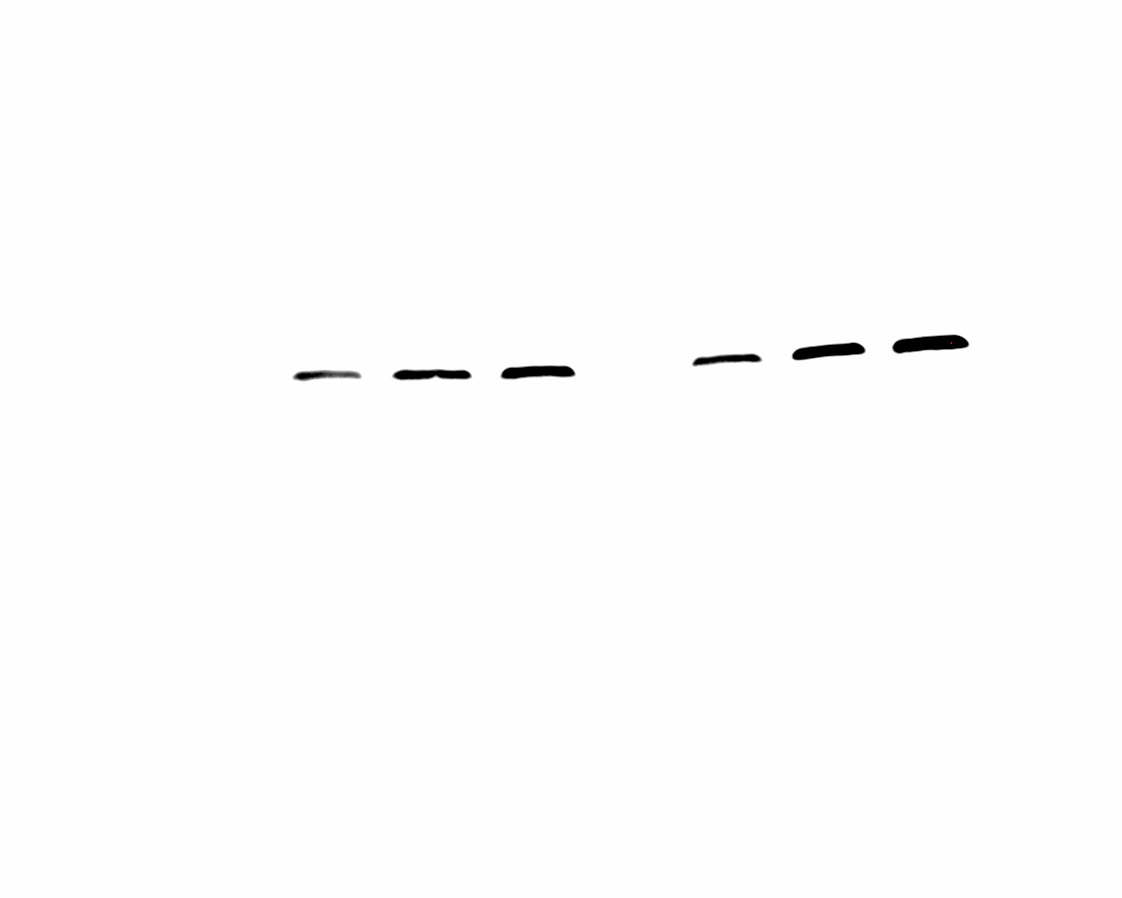

Supplement: Supplementary file 1 [file DataSheet1.ZIP › WB-mouse Bad.jpg]

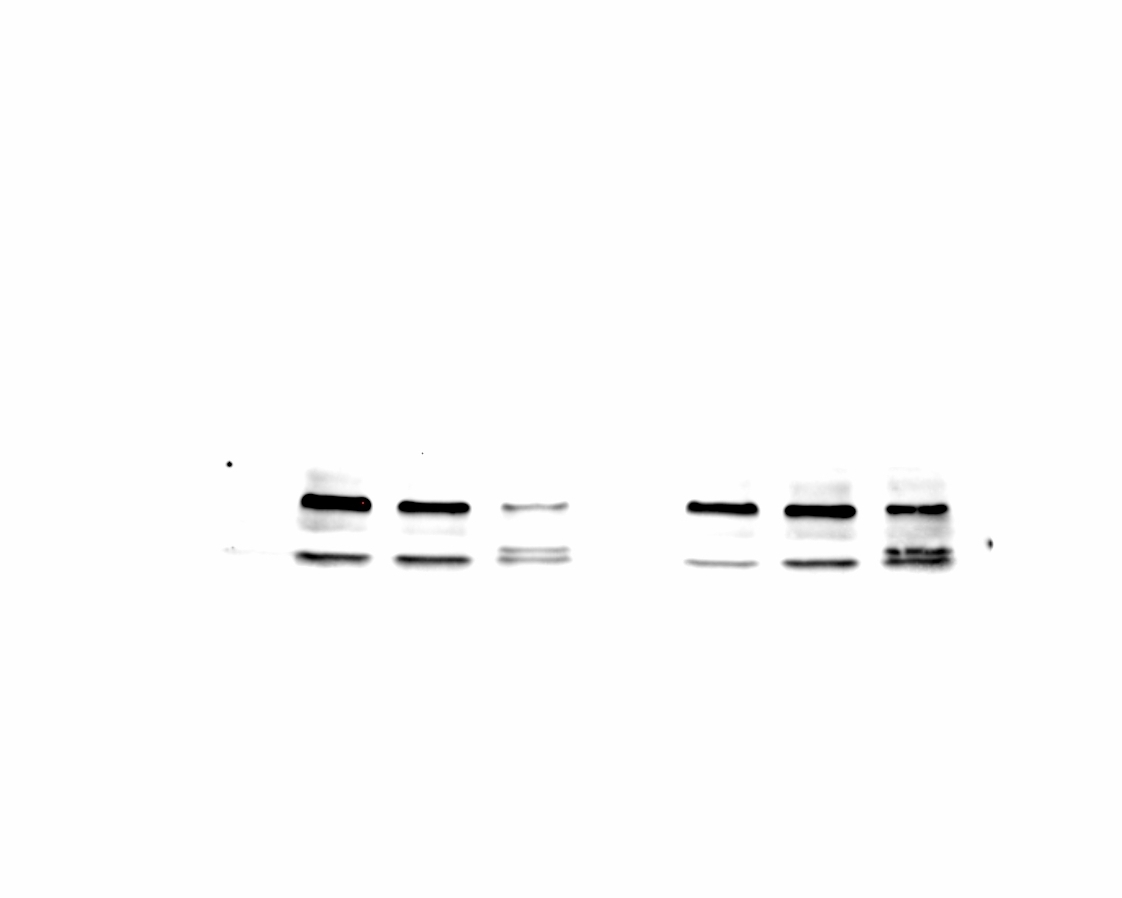

Supplement: Supplementary file 1 [file DataSheet1.ZIP › WB-mouse bcl2.jpg]

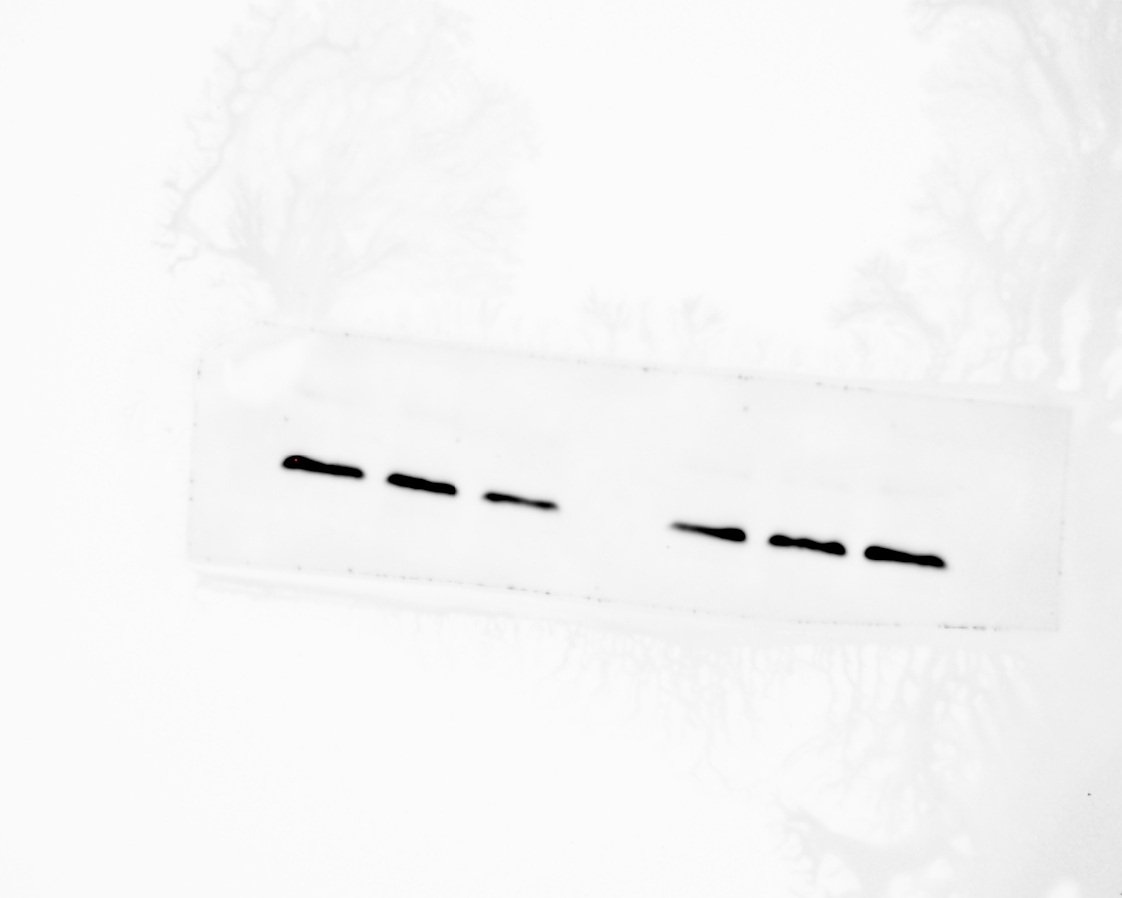

Supplement: Supplementary file 1 [file DataSheet1.ZIP › WB-mouse p-Akt.jpg]

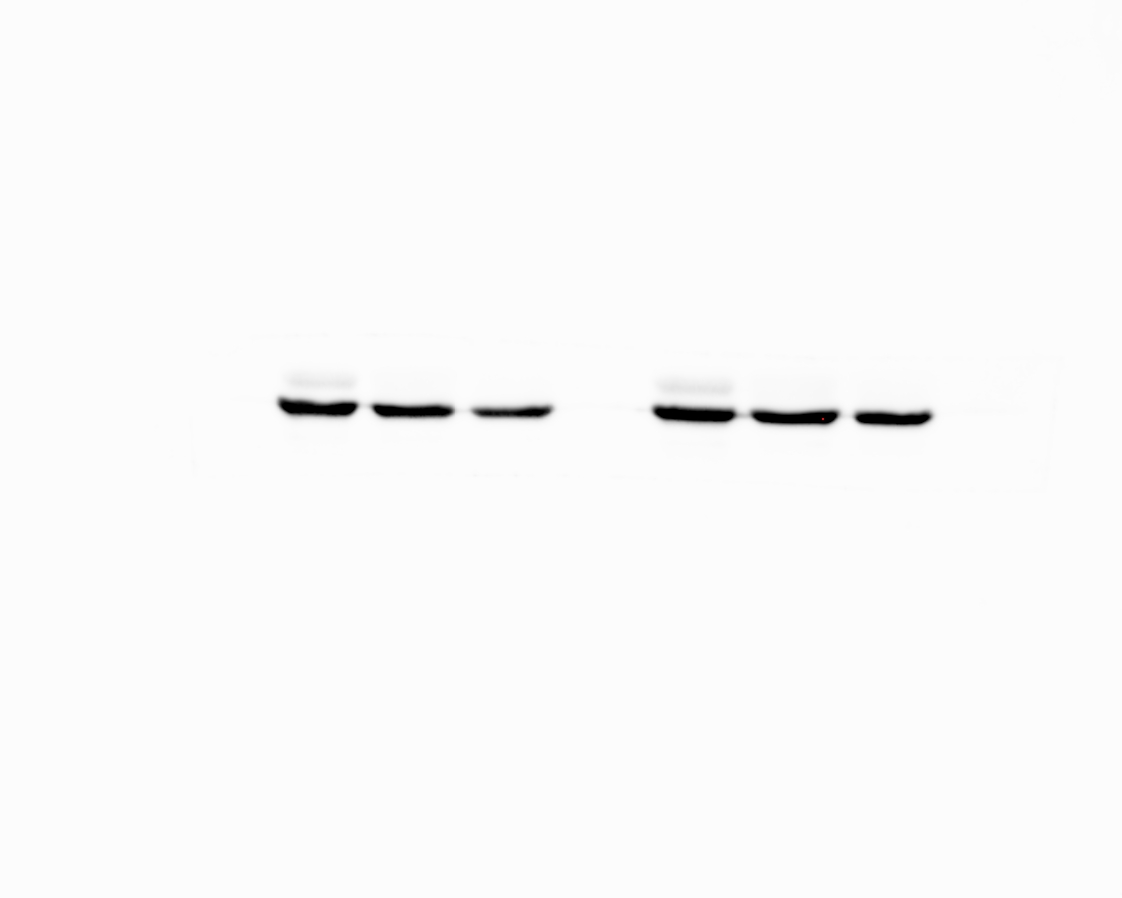

Supplement: Supplementary file 1 [file DataSheet1.ZIP › WB-mouse β-actin.jpg]

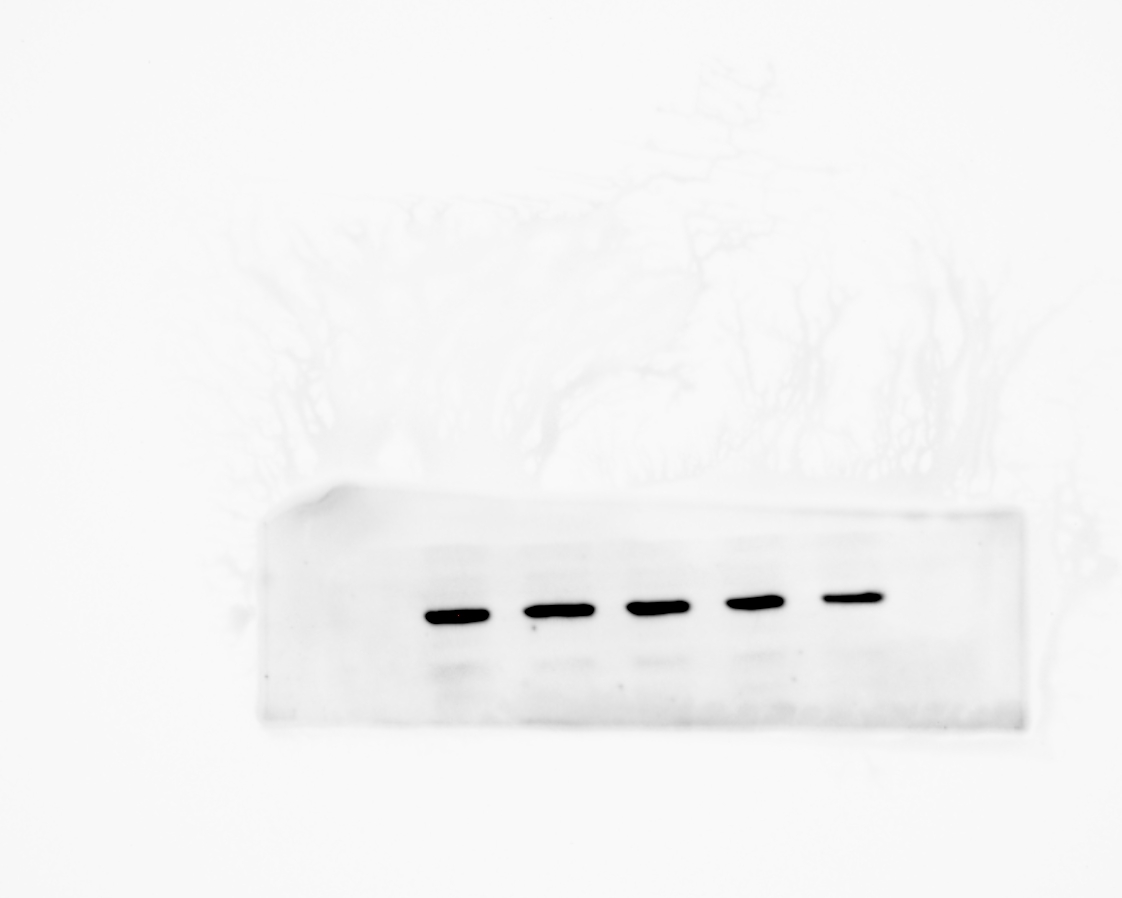

Supplement: Supplementary file 1 [file DataSheet1.ZIP › WB-panc1 Akt.jpg]

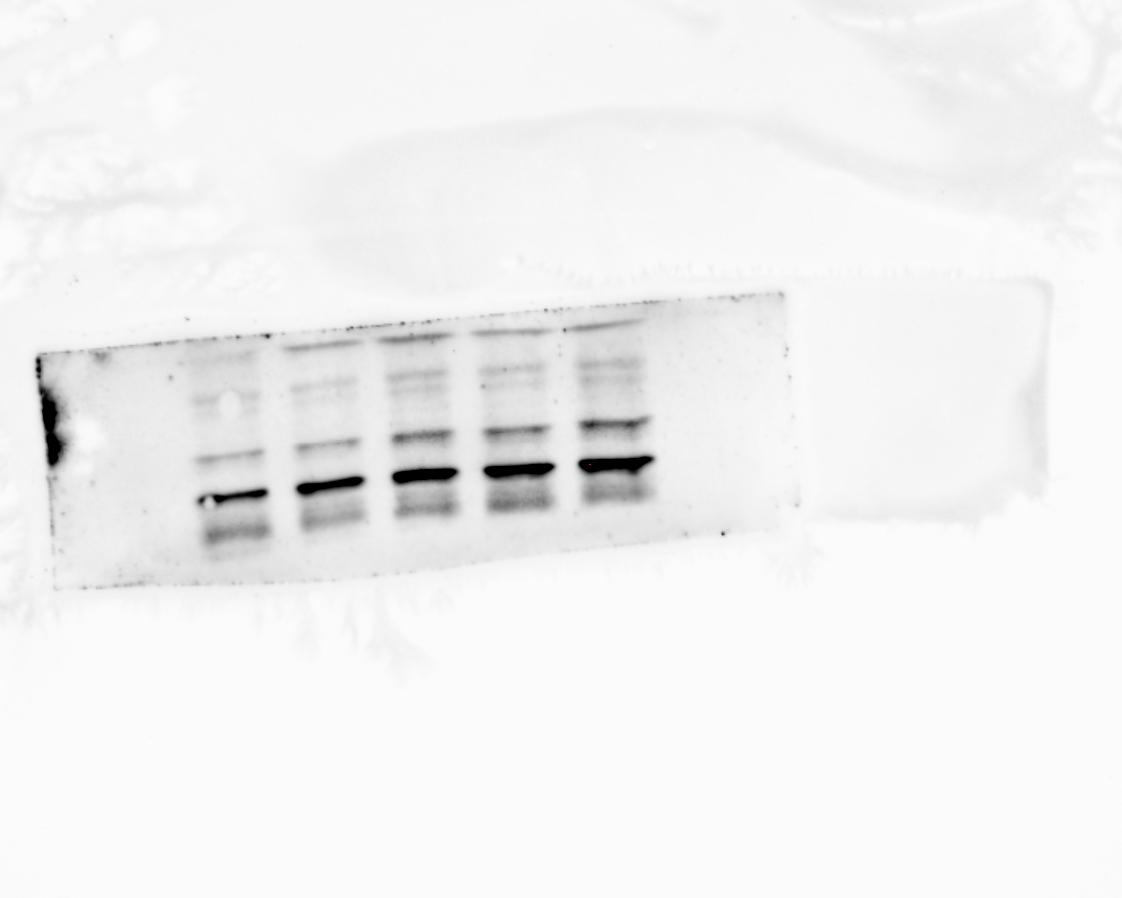

Supplement: Supplementary file 1 [file DataSheet1.ZIP › WB-panc1 bad.jpg]

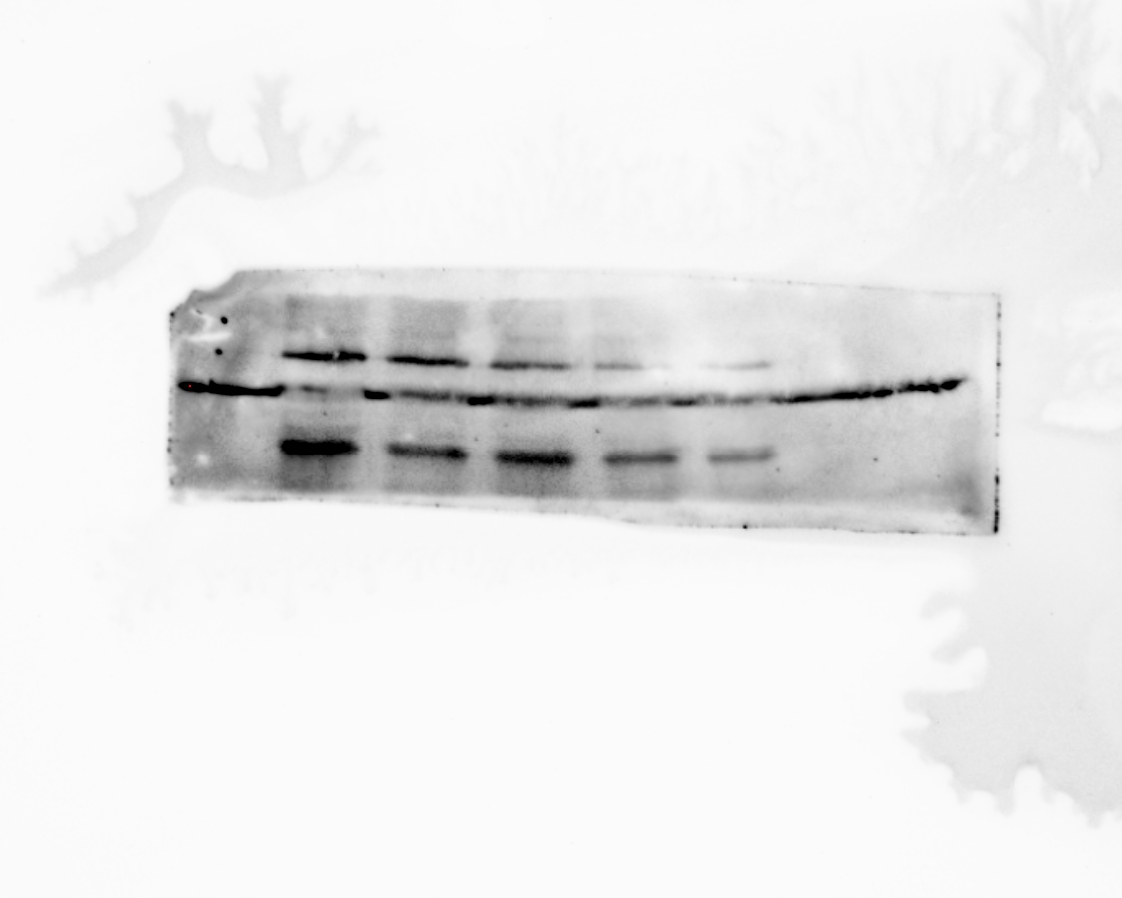

Supplement: Supplementary file 1 [file DataSheet1.ZIP › WB-panc1 bcl-2.jpg]

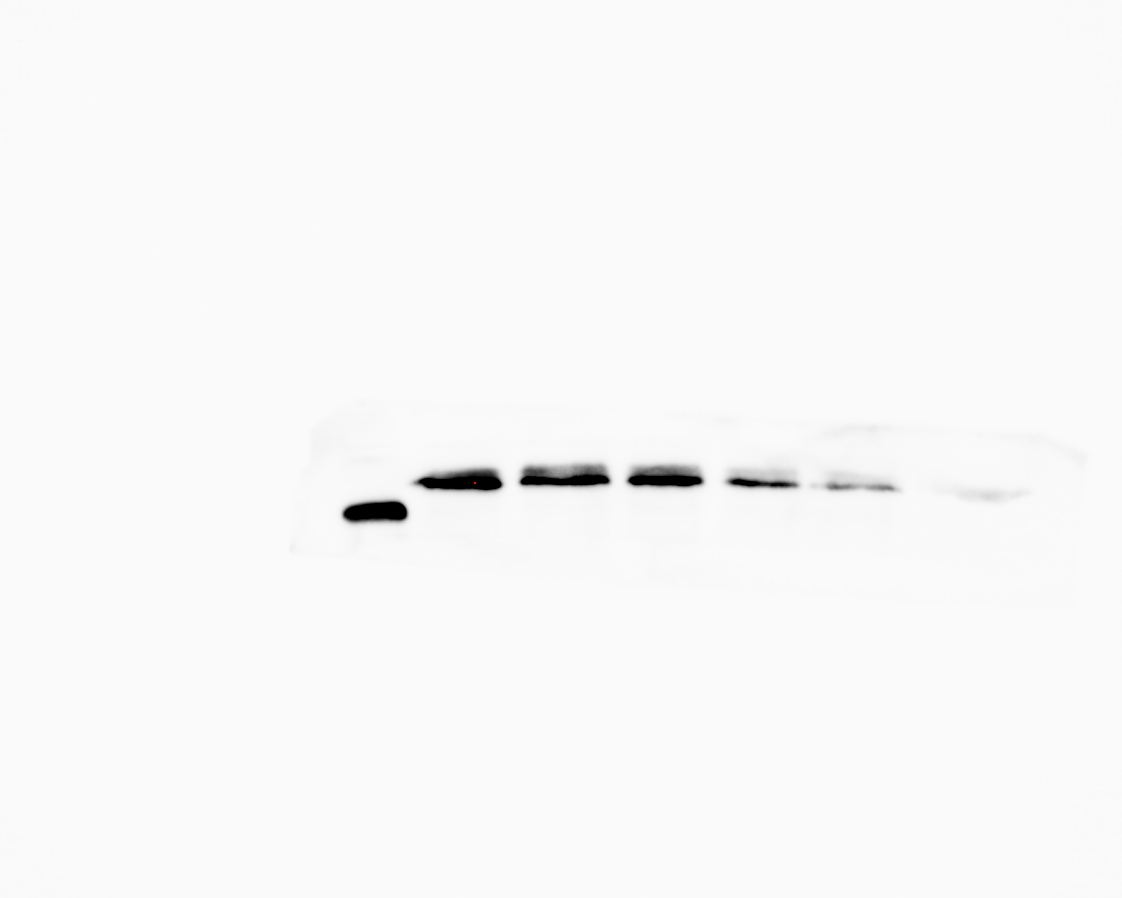

Supplement: Supplementary file 1 [file DataSheet1.ZIP › WB-panc1 pAkt.jpg]

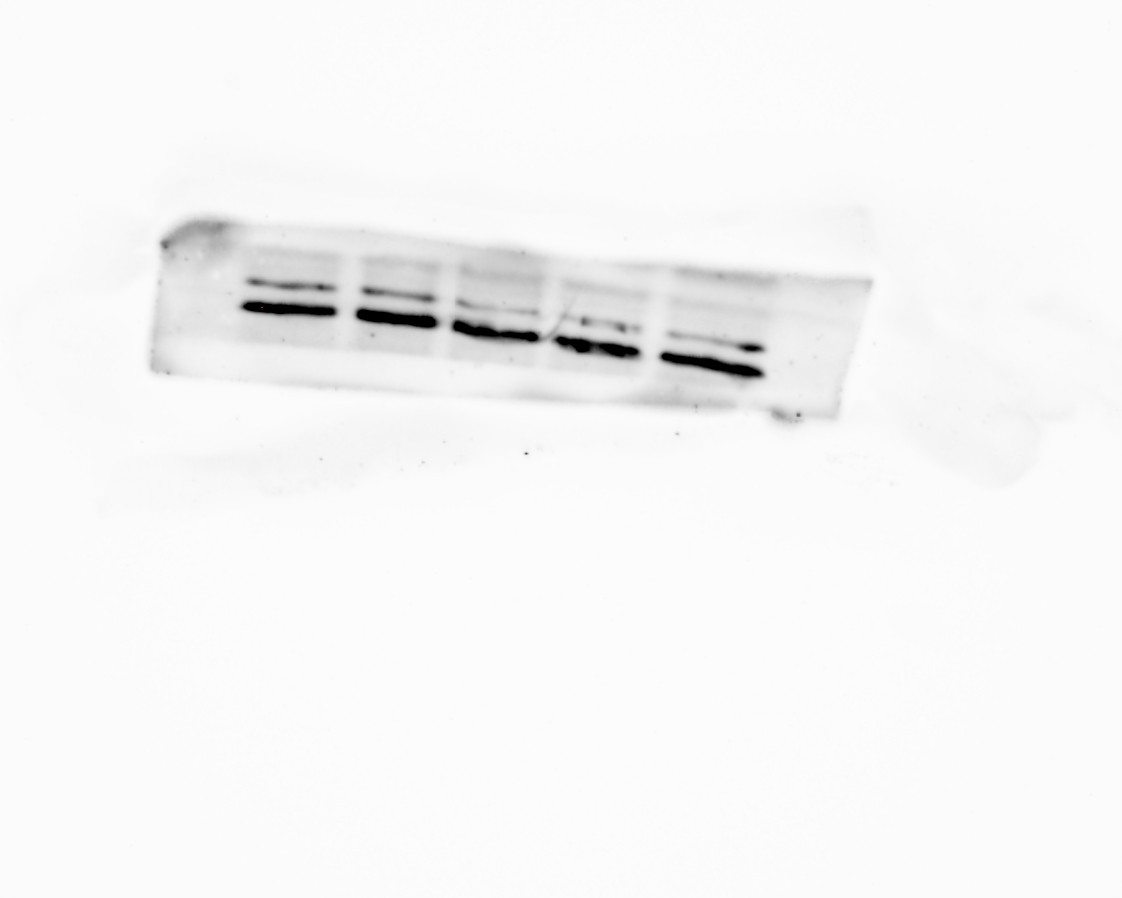

Supplement: Supplementary file 1 [file DataSheet1.ZIP › WB-panc1 β-actin.jpg]
